# Design and Synthesis of Potential Multi-Target Antidepressants: Exploration of 1-(4-(7-Azaindole)-3,6-dihydropyridin-1-yl)alkyl-3-(1*H*-indol-3-yl)pyrrolidine-2,5-dione Derivatives with Affinity for the Serotonin Transporter

**DOI:** 10.3390/ijms252011276

**Published:** 2024-10-20

**Authors:** Martyna Z. Wróbel, Andrzej Chodkowski, Agata Siwek, Grzegorz Satała, Andrzej J. Bojarski, Maciej Dawidowski

**Affiliations:** 1 Department of Drug Technology and Pharmaceutical Biotechnology, Faculty of Pharmacy, Medical University of Warsaw, 1 Banacha Street, 02-097 Warszawa, Poland; andrzej.chodkowski.tsl@gmail.com (A.C.); maciej.dawidowski@wum.edu.pl (M.D.); 2 Department of Pharmacobiology, Faculty of Pharmacy, Jagiellonian University Medical College, 9 Medyczna Street, 30-688 Kraków, Poland; agat.siwek@uj.edu.pl; 3 Department of Medicinal Chemistry, Maj Institute of Pharmacology, Polish Academy of Sciences, 12 Smetna Street, 31-343 Kraków, Poland; satala@if-pan.krakow.pl (G.S.); bojarski@if-pan.krakow.pl (A.J.B.)

**Keywords:** drug design and discovery, antidepressant drugs, multi-target directed ligands, 5-HT_1A_ receptor ligands, serotonin reuptake inhibitors, triple reuptake inhibitors

## Abstract

We describe the design, synthesis and structure–activity relationship of a novel series of 1-(4-(7-azaindole)-3,6-dihydropyridin-1-yl)alkyl-3-(1*H*-indol-3-yl)pyrrolidine-2,5-dione derivatives with combined effects on the serotonin (5-HT_1A_) and dopamine (D_2_) receptors and the serotonin (5-HT), noradrenaline (NA), and dopamine (DA) transporters as multi-target directed ligands for the treatment of depression. All of the tested compounds demonstrated good affinity for the serotonin transporter (SERT). Among them, compounds **11** and **4** emerged as the lead candidates because of their promising pharmacological profile based on in vitro studies. Compound **11** displayed a high affinity for the 5-HT_1A_ (*K*_i_ = 128.0 nM) and D_2_ (*K*_i_ = 51.0 nM) receptors, and the SERT (*K*_i_ = 9.2 nM) and DAT (*K*_i_ = 288.0 nM) transporters, whereas compound **4** exhibited the most desirable binding profile to SERT/NET/DAT among the series: *K*_i_ = 47.0 nM/167.0 nM/43% inhibition at 1 µM. These results suggest that compounds **4** and **11** represent templates for the future development of multi-target antidepressant drugs.

## 1. Introduction

Depression is one of the most severe mental disorders of our time. According to the World Health Organization, around 280 million people worldwide suffer from it, which accounts for approximately 3.8% of the population [1]. The monoamine hypothesis of depression is associated with the dysfunction of neurotransmitters such as serotonin (5-HT), noradrenaline (NA), and dopamine (DA) in the central nervous system. Alterations in the synaptic transmission of these neurotransmitters are implicated in the development of core depressive symptoms such as mood disorders, anhedonia and others [2]. Furthermore, the most commonly prescribed classes of antidepressants target these neurotransmitter systems, including monoamine oxidase A (MAO-A) inhibitors, selective serotonin reuptake inhibitors (SSRIs), and norepinephrine reuptake inhibitors (NRIs). Unfortunately, the STAR*D trial revealed that only one third of patients achieved remission with the available antidepressant therapies [3]. These results show how important is to find more effective and safer pharmacotherapy. Recent research indicates that compounds that target multiple monoaminergic pathways are most likely to be effective in treating major depressive disorder (MDD) [4,5].

**Figure 1:**
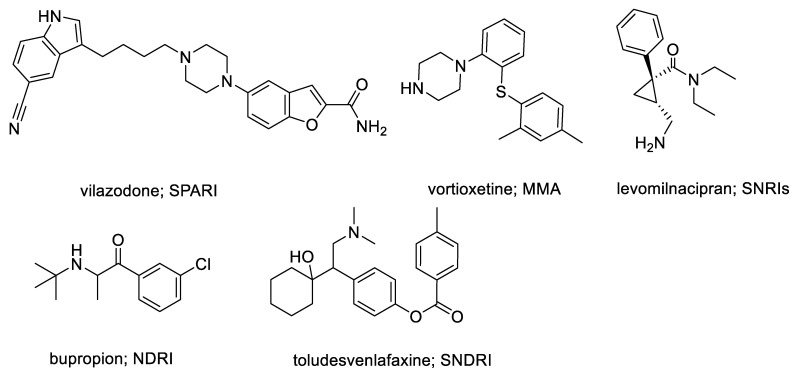
Chemical structures of known ADDs with multiple monoaminergic targets.

**Figure 2:**
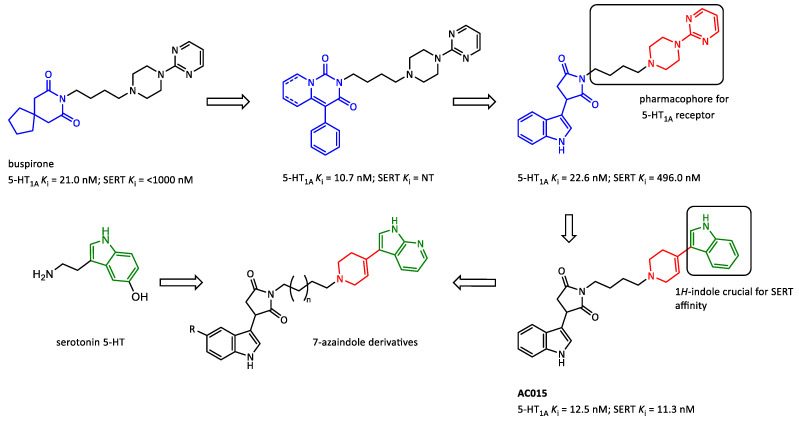
Our design strategy for 3-(1*H*-indol-3-yl)pyrrolidine-2,5-dione derivatives as potential antidepressants with double binding to the 5-HT_1A_ receptor and SERT.

One therapeutic strategy is to combine agents that target monoamine transporters (MATs) and receptors. Compounds acting as 5-HT_1A_ receptor ligands and simultaneously inhibiting the serotonin transporter (SERT) have been of particular interest to researchers, leading to the development and approval of several antidepressant drugs (ADDs). Notably, novel ADDs such as vilazodone (Figure 1), a selective partial agonist and reuptake inhibitor (SPARI), and vortioxetine (Figure 1), a multimodal antidepressant (MMA), exemplify this approach [6]. Co-administration of an SSRI and a 5-HT_1A_ autoreceptor antagonist such as pindolol or WAY-100635, has been shown to accelerate and potentiate the antidepressant effects of SSRIs, by preventing pre- or postsynaptic adaptive mechanisms of monoamine neurotransmission [7]. Other studies have explained this effect, revealing that the antagonists facilitate the normalisation of serotonergic neuron firing rates through the desensitisation of 5-HT_1A_ autoreceptors [8]. Moreover, co-administration of an SSRI and a 5-HT_1A_ receptor agonist/partial agonist causes a rapid increase in neurotransmission in the serotonergic system via postsynaptic receptor stimulation [9]. Other 5-HT_1A_ receptor partial agonists may provide additional therapeutic benefits, particularly in addressing anxiety symptoms, supported by the approval of buspirone (Figure 2) for the treatment of generalised anxiety disorder. In addition, 5HT_1A_ partial agonism may mitigate common side effects associated with SSRIs, namely gastrointestinal upset and sexual dysfunction [10].

In the ongoing research for effective pharmacotherapies for depression, increasing attention is being given to the dopaminergic system, which is involved in regulating various functions such as mood, motivation, and perception [11]. D_2_ receptors, prominently expressed in brain regions like the nucleus accumbens (NAc) and prefrontal cortex (PFC), are crucial for regulating DA release and feedback mechanisms. Moreover, dopamine receptors, along with their heterodimers, play a pivotal role in the communication and integration of various neural circuits, many of which are thought to be implicated in the pathophysiology of depression [11].

The interaction between SERT and D_2_ receptor modulation affects synaptic plasticity, particularly in stress responses and depression models. SERT plays a key role in regulating 5-HT levels in the synaptic cleft by facilitating its reuptake into presynaptic neurons. Inhibition of SERT leads to increased synaptic 5-HT, which not only enhances serotonergic neurotransmission but also indirectly modulates dopaminergic pathways. Specifically, 5-HT receptors, such as 5-HT_1A_, 5-HT_1B_, 5-HT_2A_, 5-HT_2C_ and 5-HT_3_, are involved in facilitating dopamine release in key brain regions like the prefrontal cortex and nucleus accumbens, contributing to mood regulation and reward processing [12].

Additionally, the interaction between serotonin and dopamine systems is important for the effectiveness of antidepressants [13]. Compounds that simultaneously target SERT and 5-HT_1A_ receptors enhance serotonergic activity while also modulating dopamine release. Furthermore, co-targeting D_2_ receptors can amplify this synergistic effect, particularly in the mesocorticolimbic system, thereby strengthening the antidepressant response. Drugs such as aripiprazole (a partial agonist of 5-HT_1A_, D_2_, and D_3_ receptors; 5-HT_2A_ receptor antagonist; and SERT inhibitor), brexpiprazol (a partial agonist of 5-HT_1A_, D_2_, and D_3_ receptors and antagonist of 5-HT_2A_, 5-HT_2B_, and 5-HT_7_ receptors) and cariprazine (a partial agonist of 5-HT_1A_, D_2_, and D_3_ receptors and 5-HT_2A_ receptor antagonist) modulate both serotonergic and dopaminergic neurotransmission, thereby exerting antidepressant effects [14,15]. Therefore, the multi-target approach explored in our work leverages these synergistic interactions.

Another important therapeutic strategy for treatment-resistant depression is to combine agents that target multiple MATs, especially SERT, the norepinephrine transporter (NET), and the dopamine transporter (DAT) [16]. Levomilnacipran (Figure 1), approved in 2013, is the latest serotonin–noradrenaline reuptake inhibitor (SNRI) with a unique pharmacological profile and demonstrates approximately two-fold higher potency for NET than for SERT. Bupropion (Figure 1), classified as a dual-reuptake inhibitor—specifically, a norepinephrine and dopamine reuptake inhibitor (NDRI)—has also been approved to treat MDD [16]. Evidence from animal studies suggests that bupropion can enhance the efficacy of other antidepressants. When co-administered intraperitoneally to mice at subactive doses along with inactive doses of other ADDs (including paroxetine, fluvoxamine, venlafaxine, and milnacipran), the immobility time in the forced swimming test (FST) was reduced. This finding indicates that NDRIs may enhance the efficacy of SSRIs and SNRIs, a view that is consistent with the preliminary clinical evidence [17]. Recent research has highlighted that triple-reuptake inhibitors (TRIs or serotonin–norepinephrine–dopamine reuptake inhibitors (SNDRIs)) are promising chemical entities for the treatment of depression. Several of them are now in different stages of clinical trials. For example, toludesvenlafaxine (also formerly known as ansofaxine, Figure 1) was the first SNDRI approved in China (2022) and is currently under investigation by the Food and Drug Administration [18].

Focusing our interest on the design and development of multi-target directed ligands (MTDLs) for the treatment of depression, we employed the concept of molecular hybridisation, which involves merging two or more molecular scaffolds into a single molecule [2]. We identified compound **AC015** (Figure 2), a lead with dual affinity towards SERT and the 5-HT_1A_ receptor [19]. We designed this compound using buspirone as a template, which belongs to long-chain arylpiperazines (LCAPs), established 5-HT_1A_ ligands. In the initial step of this redesign, we replaced the 8-azaspiro[4.5]decane-7,9-dione moiety with pyrido[1,2-*c*]pyrimidines [20], and subsequently with 3-(1*H*-indol-3-yl)pyrrolidine-2,5-dione [19], while retaining the aliphatic linker important for binding to the 5-HT_1A_ receptor and SERT (Figure 2). Simultaneously, we replaced the arylpiperazine residue with 1,2,3,6-tetrahydropyridinylindoles [21], which are known for their affinity for SERT and the 5-HT_1A_ receptor [22,23]. Continuing our examination on the group of 3-(1*H*-indol-3-yl)pyrrolidine-2,5-diones with double 5-HT_1A_ receptor/SERT binding affinity [19], we opted to modify the indole system within the SERT pharmacophore of the molecule. So far, we have studied the effect of introducing various electron-donating and electron-accepting substituents in the C5 position of the indole of the 3-(1,2,3,6-tetrahydropyridin-4-ly)-1*H*-indole part and in the 3-(1*H*-indol-3-yl)pyrrolidine-2,5-dione part [21,24] of the molecule, as well as the impact of the presence or absence of a double bond in the 3-piperidin-4-yl-1*H*-indole ring [24].

Azaindole derivatives are known for their significant biological activity; they are used as convenient bioisosteres of indole and purine systems [25,26]. Azaindoles exist as four positional isomers: 4-, 5-, 6-, and 7-azaindole [26]. Substituting an indole core with azaindole is a strategic approach to modulate the key properties of a compound such as a solubility, *pK_a_*, and lipophilicity; binding to a molecular target; and the ADME-tox profile. Thus, this modification is a valuable tool in the drug discovery process [27,28]. Importantly, studies indicate the potential of 1*H*-pyrrolo[2,3-*b*]pyridines (7-azaindoles) as ligands for the dopamine D_2_ and D_4_ receptors [29] and the 5-HT_6_/5-HT_2A_ receptors [30]. Moreover, 3-(1,2,3,6-tetrahydropyridin-4-yl)-1*H*-pyrrolo[2,3-*b*]pyridines have been investigated as antiseizure agents [31]. Additionally, through the use of MAT three-dimensional (3D) protein models and structure-based computational tools, 3-(1,2,3,6-tetrahydropyridin-4-ly)-1*H*-indole derivatives have been discovered as MAT ligands [30]. This suggests that its bioisostere 3-(1,2,3,6-tetrahydropyridin-4-yl)-1*H*-pyrrolo[2,3-*b*]pyridines derivatives may also exhibit similar SNDRI activity.

Keeping in mind the concept of developing MTDLs with a high dual affinity for the 5-HT_1A_ receptor and SERT, we conducted a new exploratory study focused on the replacement of the 3-(1,2,3,6-tetrahydropyridin-4-ly)-1*H*-indole moiety with 3-(1,2,3,6-tetrahydropyridin-4-yl)-1*H*-pyrrolo[2,3-*b*]pyridine (Figure 2). According to Mewshaw et al. [32], such replacement in the pharmacophore part of the molecule can potentially increase its binding affinity for the 5-HT_1A_ receptor and its selectivity over the α_1_-adrenoceptor [26]. In our workflow, we evaluated the synthesised derivatives for their 5-HT_1A_ receptor affinity and serotonin reuptake inhibition. Subsequently, we tested selected compounds for their binding to the D_2_, 5-HT_2A_, 5-HT_6_, and 5-HT_7_ receptors, as well as their affinity for DAT and NET. We performed these assays to assess whether the designed compounds exhibit an improved therapeutic profile by targeting multiple monoamine receptors and transporters, thereby enhancing their potential effectiveness as multi-target ADDs.

## 2. Results and Discussion

### 2.1. Chemistry

**Scheme 1:**
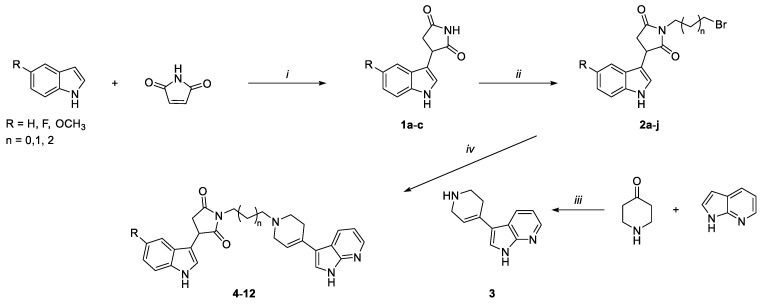
The synthesis pathways of the investigated compounds 3-(1*H*-indol-3-yl)pyrrolidine-2,5-dione derivatives **4**–**12**. Reagents and conditions: (*i*) CH_3_COOH, 118 °C; (*ii*) BrCH_2_(CH_2_)_n_CH_2_Br, K_2_CO_3_, acetone, 60 °C; (*iii*) CH_3_OH_dry_, CH_3_ONa, 65 °C; (*iv*) K_2_CO_3_, KI_(cat.)_, CH_3_CN, 85 °C.

The synthetic route for designed compounds is presented in Scheme 1. The first-stage condensation of maleimide with 1*H*-indole in the presence of glacial acetic acid resulted in the formation of products **1a**–**c** [33,34]. Next, we subjected the respective derivatives **1a**–**c** to *N*-alkylation with 1,4-dibromobutane, 1,3-dibromopropane, or 1,2-dibromoethane at the imide N1 position. This yielded the corresponding 3-(1*H*-indol-3-yl)pyrrolidine-2,5-dione **2a**–**c**, 3-(5-methoxy-1*H*-indol-3-yl)pyrrolidine-2,5-dione **2d**–**f**, or 3-(5-fluoro-1*H*-indol-3-yl)pyrrolidine-2,5-dione **2g**–**i** derivatives (Scheme 1), following a previously developed synthesis method [19,24]. We obtained 3-(1,2,3,6-tetrahydropyridin-4-yl)-1*H*-pyrrolo[2,3-*b*]pyridine (**3**) in a one-step synthesis with a variation of a described procedure [32] that consisted of condensation of 1*H*-pyrrolo[2,3-*b*]pyridine with 4-piperidone under an inert atmosphere (argon) (Scheme 1). We synthesised the target compounds **4**–**12** via *N*-alkylation of compounds **2a**–**i** with 3-(1,2,3,6-tetrahydropyridin-4-yl)-1*H*-pyrrolo[2,3-*b*]pyridine (**3**), according to previously described procedures [19]. We purified all of the final compounds using column chromatography and converted them into their water-soluble hydrochloride salts.

### 2.2. In Vitro Receptor Assay and Structure–Activity Relationship (SAR)

#### 2.2.1. In Vitro Tests for the 5-HT_1A_ Receptor and SERT

We tested synthesised target compounds **4**–**12** for their inhibition of serotonin reuptake and their binding affinity for the 5-HT_1A_ receptor. Specifically, we determined the 5-HT_1A_ receptor and SERT binding affinity by investigating the displacement of [^3^H]-8-OH-DPAT and [^3^H]-imipramine to CHO-K1 and HEK-293 cell membrane homogenates, respectively. To evaluate the impact of incorporating 7-azaindole in place of the 1*H*-indole moiety within the tested derivatives on receptor and transporter affinity, we also included binding data for the 5-HT_1A_ receptor and SERT from the previously described 1*H*-indole analogues **13**–**21** [19,21] of compounds **4**–**12**. The results are summarised in Table 1.

Compounds **4**–**12** displayed a good to moderate affinity for SERT (*K*_i_ = 9.2–254.0 nM). We observed the highest SERT inhibition for compounds **4** (*K*_i_ = 47.0 nM), **8** (*K*_i_ = 23.0 nM), and **11** (*K*_i_ = 9.2 nM); however, in general, the 7-azaindole derivatives showed a loss of affinity for the 5-HT_1A_ receptor (except for **10**, with *K*_i(5-HT1A)_ = 128.0 nM) compared with their 1*H*-indole counterparts **13**–**21**. 

When analysing the binding results for 7-azaindoles **4**–**12**, we observed that the inclusion of a three-methylene spacer had a positive impact on SERT affinity (compounds **8** and **11**), consistent with the results obtained for the series of 1*H*-indole analogues **13**–**21** (excluding compound **17**). Generally, in the series 3-(1*H*-indol-3-yl)pyrrolidine-2,5-diones reported by us so far [19,21], the presence of a three-methylene linker is favourable for SERT binding. The replacement of the 1*H*-indole residue with a 1*H*-pyrrolo[2,3-*b*]pyridine moiety did not alter this beneficial effect of the linker length. However, when comparing 1*H*-indoles **13**–**21** with their newly obtained respective analogues **4**–**12**, we concluded that the introduction of a 1*H*-pyrrolo[2,3-*b*]pyridine moiety leads to a slight decrease in SERT binding.

Next, we analysed the impact of the 1*H*-indole substituent (R = H, F, or OCH_3_) in the 3-(1*H*-indol-3-yl)pyrrolidine-2,5-dione fragment on 5-HT_1A_ receptor and SERT binding. Among compounds **4**–**12**, derivative **10** (R = F) exhibited the best affinity for the 5-HT_1A_ receptor. Notably, this compound demonstrated significantly higher affinity for the 5-HT_1A_ receptor than the other compounds from the investigated 7-azaindole series (its *K*_i_ value was >5 times lower than for other compounds in the series). When analysing the effect of the 1*H*-indole substituent on the affinity for SERT, we observed a similar pattern as for the 5-HT_1A_ receptor, with an increase in SERT affinity for compounds with R = OCH_3_ or F. Specifically, compound **8** (R = OCH_3_) with a SERT *K*_i_ of 23.0 nM and compound **11** (R = F) with a SERT *K*_i_ of 9.2 nM showed enhanced affinity compared with compound **4**, where R = H (SERT *K*_i_ = 47.0 nM).

The substitution at the indole C-5 position proved advantageous, leading to the highest 5-HT_1A_ receptor and SERT binding affinities within the series: compound **10** showed a *K*_i_ of 128 nM for the 5-HT_1A_ receptor, and compound **11** showed a *K*_i_ of 9.2 nM for SERT. These findings are consistent with previously reported results, supporting the beneficial effects of substitutions at the indole C-5 position in the series of 3-(1*H*-indol-3-yl)pyrrolidine-2,5-diones on dual 5-HT_1A_ receptor and SERT binding [21].

When we compared the 5-HT_1A_ receptor or SERT affinity results of the best compounds—**8**, **10**, and **11**—with their respective 1*H*-indole analogues **17** (R = OCH_3_), **19** (R = F), or **20** (R = F), we observed a decrease in the 5-HT_1A_ receptor and SERT affinities. This suggests that while the substitution at the R position can enhance 5-HT_1A_ receptor or SERT binding and a three-methylene linker is favourable for SERT in the explored pyrrolidine-2,5-dione derivatives **4**–**12**, the introduction of a 1*H*-pyrrolo[2,3-*b*]pyridine moiety in these derivatives generally results in lower overall affinity for SERT compared with the indole-based analogues **13**–**21** and an overall loss of their affinity for the 5-HT_1A_ receptor. It is worth mentioning that the results we obtained for the explored series of pyrrolidine-2,5-dione derivatives do not align with findings reported by others regarding the impact of replacing the indole moiety with a 7-azaindole residue. Zhou et al. [35] and Mewshaw et al. [32] demonstrated that such a replacement generally results in the loss or decrease in affinity for SERT while maintaining potent binding affinity for the 5-HT_1A_ receptor.

#### 2.2.2. In Vitro Tests for the D_2L_, 5-HT_6_, 5-HT_7_, 5-HT_2A_, and α_1_ Adrenoceptors, as Well as DAT and NET

Keeping in mind the concept of designing multi-target antidepressants, due to their high affinity for SERT, we selected compounds **4**, **8**, **11**, and **20** (the indole-based analogue of compound **11**) for follow-up multi-receptor binding assays. An additional rationale behind our selection was the findings reported by Staroń et al. [36]: 4-(5-{[(2-{5-fluoro-1*H*-pyrrolo[2,3-*b*]pyridin-3-yl}ethyl)amino]methyl}furan-2-yl)phenol (compound **42** from that study) demonstrated affinity for the 5-HT_6_ and 5-HT_2A_ receptors and was active in vivo, in a procognitive animal model. Next, we evaluated compounds **4**, **6**, **8**, **9**, **11**, and **12** for their binding affinity to MATs. We conducted these assays to assess whether compounds within the series of 3-(1*H*-indol-3-yl)pyrrolidine-2,5-diones incorporating the 7-azaindole residue **4**–**12** could exhibit an improved therapeutic profile by targeting multiple receptors and transporters, thereby enhancing their potential effectiveness as antidepressants. To consider potential cardiovascular side effects, we also evaluated compounds **4**, **6**, **8**, **9**, and **12** for their binding affinity to the α_1_-adrenoceptor.

To determine the multi-receptor binding affinity profile of compounds **4**, **8**, and **11** with the highest affinity for SERT among the obtained derivatives, we performed radioligand binding assays for the cloned human D_2_L, 5-HT_6_, and 5-HT_7_ receptors, and for the native 5-HT_2A_ receptor. Specifically, we assessed the displacement of [^3^H]-raclopride, [^3^H]-LSD, and [^3^H]-5-CT from the cloned human D_2_L, 5-HT_6_, and 5-HT_7_ receptor, respectively, stably expressed in HEK293 cells, and [^3^H]-ketanserin from the 5-HT_2A_ receptor from the rat cortex. The results are summarised in Table 2.

In the multi-receptor binding study, compounds **4**, **8**, and **11** exhibited low affinity for the 5-HT_2A_, 5-HT_6_, and 5-HT_7_ receptors. Compound **11** was the only one to display dual-binding affinity for the 5-HT_1A_ receptor and SERT. Thus, to evaluate the impact of replacing the indole with 7-azaindole within this series, we also tested compound **20**, the indole analogue of compound **11**, in the same assay. We found that replacing 1*H*-indole with 7-azaindole resulted in a significant loss of affinity for the 5-HT_2A_, 5-HT_6_, and 5-HT_7_ receptors. Thus, the 3-(1,2,3,6-tetrahydropyridin-4-ly)-1*H*-indole moiety is crucial for maintaining binding activity to these serotonin receptors.

Interestingly, despite losing affinity to the 5-HT receptors, the tested 7-azaindoles **4**, **8**, and **11** exhibited high affinity for the D_2_ receptor (with *K*_i_ values ranging from 13.0 to 78.0 nM), which was directly evidenced by comparison of analogues **11** and **20** (Table 2). Structural modifications, such as varying the length of the alkyl chain or replacing 1*H*-indole with 7-azaindole, did not significantly affect the affinity for the D_2_ receptor. However, it appears that different R substituents do influence receptor binding affinity. Compound **8**, which has an R = OCH_3_ substituent, exhibited the highest affinity for the D_2_ receptor, with a *K*_i_ of 13.0 nM. The consistency in D_2_ receptor binding suggests that in the explored series of derivatives, the 3-(1*H*-indol-3-yl)pyrrolidine-2,5-dione part of the molecule plays a key role in the interaction with the D_2_ receptor, and this interaction is maintained regardless of the modifications of the 3-(1,2,3,6-tetrahydropyridin-4-ly)-1*H*-indole part. Such a conclusion could explain the discrepancy observed when comparing our results with those obtained for other derivative groups, where the replacement of indole with 7-azaindole led to a loss of D_2_ receptor affinity reported by Kulagowski et al. [29] and Staroń et al. [36]. In contrast, for the 3-(1*H*-indol-3-yl)pyrrolidine-2,5-dione derivatives we tested, there was no significant decrease in D_2_ receptor binding following introduction of the 7-azaindole moiety.

Additionally, we evaluated the affinity of the selected compounds for the α_1_-adrenoceptor to assess their potential cardiovascular risks. High affinity for the α_1_-adrenoceptors might lead to undesirable side effects such as hypotension. The results are summarised in Table 3. All compounds tested except compound **4** showed moderate affinity for the α_1_-adrenoceptor, which can be considered a desirable receptor profile.

Next, we determined the affinity of compounds **4**, **6**, **8**, **9**, **11**, and **12** for DAT and NET. We performed radioligand binding assays using membranes from CHO-K1 cells stably transfected with human DAT and membranes from MDCK cells stably transfected with human NET. This was accomplished by assessing the displacement of [^3^H]-WIN35,428 and [^3^H]-nisoxetine from cloned human DAT and NET, respectively. The results are summarised in Table 4. Considering the factors we discussed in the Introduction, there is a compelling rationale for the development of a single ADD capable of simultaneously enhancing the levels of 5-HT, NA, and DA. Such an approach could potentially overcome the limitations associated with single- and dual-reuptake inhibitors, offering a more comprehensive treatment option for depression [16].

The challenge in developing the therapeutic potential of SNDRIs is in achieving an optimal balance between SERT, NET, and DAT inhibition. The accurate optimal ratio between the inhibition of these three transporters is not fully explained; however, in vitro binding affinity should be in the order SERT > NET > DAT [16]. It is well established that excessive inhibition of NET can lead to adverse cardiovascular effects, while excessive inhibition of DAT can result in potential reinforcing effects, abuse liability, as well as motor stimulation and stereotypic behaviours [16]. Consequently, it is generally believed that an ideal SNDRI profile should exhibit higher inhibition of SERT than NET and moderate inhibition of DAT. The findings from recent studies suggest that an optimal receptor profile is characterised by SERT, NET, and DAT occupancy of ≥80%, 50−70%, and ≤30%, respectively, in an in vivo test. This balance is important for the development of an effective SNDRI, ensuring therapeutic benefit while reducing the risk of adverse effects [37].

Among the tested compounds, derivative **4** exhibited the highest affinity for NET, with a *K*_i_ of 167.0 nM. This may be described as a beneficial impact of the four-carbon linker, which appears to favour binding to NET. In contrast, the remaining compounds displayed weak inhibition of NET, approximately 40% at a concentration of 1 µM.

Analysing the binding affinity to DAT, we observed that compounds with a two- or three-carbon linker exhibited a higher affinity for DAT compared with compound **4**, which has a four-carbon linker. Specifically, compounds **11** and **12** demonstrated the highest affinity, with *K*_i_ values of 288.0 and 229.0 nM, respectively. Notably, compound **11** (with R = F) and compound **12** (with R = F) showed improved DAT binding compared with the analogues with R = H or OCH_3_, indicating that the introduction of a fluorine substituent enhances DAT affinity.

Considering the selectivity ratio between the tested transporters, we obtained the most desired binding profile for compound **4**, with NET/SERT = 3.5, and compound **11**, with DAT/SERT = 31.3. In summary, these results highlight that compounds **4** and **11** represent the most promising starting points for lead optimisation. They offer favourable MAT inhibition profiles that align with the desired criteria for SNDRIs. Further optimisation of these compounds could enhance their efficacy and selectivity, potentially leading to the development of effective therapeutic agents.

## 3. Materials and Methods

### 3.1. General Remarks

Commercially available chemicals were used without further purification. Thin-layer chromatography (TLC) was carried out on Merck TLC Silica gel 60 glass plates. Compounds were visualised by ultraviolet (UV) light (254 nm). The partition factors (R_f_) were calculated as the ratio of the distance migrated by the compound to the distance migrated by the CH_2_Cl_2_/MeOH (90:10 [*v*/*v*]) as an eluent. Room temperature refers to 20–25 °C. Manual flash column chromatography (CC) was performed using Merck Silica gel 60 (particle size 0.040–0.063 mm, 230–400 mesh ASTM), and automated preparative CC was performed on a Buchi Reveleris Prep purification system using linear gradient elution. Melting points were determined on an Electrothermal IA9200 apparatus (Cole-Parmer Ltd., Stone, Staffordshire, UK) with open capillary tubes and were uncorrected. The purity (>95%) and homogeneity of the compounds were routinely confirmed by ^1^H nuclear magnetic resonance (NMR) spectra, which were recorded on an INOVA 500 MHz (Varian, Palo Alto, CA, USA), an AVANCE III HD 500 MHz (Bruker BioSpin, Rheinstetten, Germany), or a 400-MR DD2 400 MHz (Agilent Technologies, Santa Clara, CA, USA) instrument in acetone-*d*_6_, CD_3_OD, or DMSO-*d*_6_. The NMR peaks are reported as follows: chemical shift (*δ*) in parts per million (ppm) relative to residual non-deuterated solvent. The resonance signals are described in the following order: multiplicity, coupling constant (in Hz), and integration. High-resolution mass spectrometry (HRMS) was performed using a Micromass LCT TOF (Waters Corporation, Milford, MA, USA) mass spectrometer equipped with an electrospray ionisation source, a time-of-flight analyser, and a microchannel plate detector. Samples of the tested compounds were prepared in methanol (at a concentration of 1 mg/L). The MS detection settings were as follows: a source temperature of 80 °C, desolvation temperature of 150 °C, desolvation gas flow rate of 200 L/h, cone gas flow of 100 L/h, capillary potential of 3.50–5.00 kV, cone potential of 26–50 V, extraction cone potential of 4–20 V, and RF Lens of 260–350 V. Nitrogen was used for nebulising and as the drying gas. The data were obtained in a scan mode ranging from 20 to 2000 *m*/*z* in 1.0 s time intervals. The data acquisition software was MassLynx V 3.5 (Waters). Intermediates **1a**–**c** and the 1-(bromoalkyl)-3-(1*H*-indol-3-yl)pyrrolidine-2,5-dione derivatives **2a**–**i** were synthesised according to previously described procedures [19,21,24]. The ^1^H NMR and ^13^C NMR and HRMS spectra of compounds **4**–**12** are available in the Supplementary Materials.

#### 3.1.1. Procedure for the Synthesis of 3-(1,2,3,6-Tetrahydropyridin-4-yl)-1*H*-pyrrolo[2,3-*b*]pyridine (**3**)

Starting compound **3** was obtained according to previously described procedures [32].

#### 3.1.2. General Procedure for the Synthesis of the 3-(1*H*-Indol-3-yl)pyrrolidine-2,5-dione Derivatives **4**–**12**

A mixture of appropriate derivatives of 1-(4-bromoalkyl)-3-(5-substituted-1*H*-indol-3-yl)pyrrolidine-2,5-dione **2a**–**i** (0.5 mmol), 3-(1,2,3,6-tetrahydropyridin-4-yl)-1*H*-pyrrolo[2,3-*b*]pyridine (**3**) (0.5 mmol), K_2_CO_3_ (1.0 mmol), and a catalytic amount of KI with 35 mL of acetonitrile were stirred at reflux for 4–5 h. The reaction time was monitored using TLC. After cooling, the mixture was filtered, and the filtrate was evaporated to dryness. The crude residue was purified by manual or automated flash chromatography using CH_2_Cl_2_/MeOH (98:2, then 97:3, then 95:5 [*v*/*v*]) as an eluent. Proper fractions were identified using TLC and evaporated to dryness to yield analytically pure compounds **4**–**12**.

##### Synthesis of 1-(4-(4-(1*H*-Pyrrolo[2,3-*b*]pyridin-3-yl)-3,6-dihydropyridin-1(2*H*)-yl)butyl)-3-(1*H*-indol-3-yl)pyrrolidine-2,5-dione (**4**)

The title compound was isolated as a yellow solid. Yield: 48%; m.p. 172–177 °C; R_f_ = 0.17; ESI HRMS (*m*/*z*): calcd for C_28_H_30_N_5_O_2_^+^ [M + H]^+^ 468.2400, found: 468.2406; ^1^H NMR (501 MHz, acetone-*d*_6_) δ 10.71 (s, 1H), 10.25 (s, 1H), 8.26–8.20 (m, 2H), 7.54–7.45 (m, 2H), 7.42 (dt, *J* = 8.2, 1.0 Hz, 1H), 7.34 (dd, *J* = 2.6, 0.8 Hz, 1H), 7.16–7.06 (m, 2H), 7.04 (ddd, *J* = 8.0, 7.0, 1.0 Hz, 1H), 6.19 (tt, *J* = 3.6, 1.6 Hz, 1H), 4.42 (ddd, *J* = 9.5, 4.9, 0.8 Hz, 1H), 3.59 (t, *J* = 7.0 Hz, 2H), 3.32 (dd, *J* = 18.0, 9.5 Hz, 1H), 3.14 (q, *J* = 2.7 Hz, 2H), 2.92 (s, 10H), 2.86 (dd, *J* = 18.0, 4.9 Hz, 1H), 2.67 (t, *J* = 5.7 Hz, 2H), 2.60–2.53 (m, 2H), 2.48 (t, *J* = 7.1 Hz, 2H), 1.76–1.64 (m, 2H), 1.62–1.53 (m, 2H); ^13^C NMR (126 MHz, acetone-*d*_6_) δ 179.0, 177.2, 150.6, 144.0, 138.1, 130.6, 129.3, 127.3, 123.8, 123.2, 122.7, 120.1, 119.8, 119.6, 118.4, 116.7, 116.7, 112.8, 112.7, 58.6, 53.9, 51.3, 39.3, 39.0, 37.2, 29.4, 26.5, 25.2.

##### Synthesis of 1-(3-(4-(1*H*-Pyrrolo[2,3-*b*]pyridin-3-yl)-3,6-dihydropyridin-1(2*H*)-yl)propyl)-3-(1*H*-indol-3-yl)pyrrolidine-2,5-dione (**5**)

The title compound was isolated as a yellow solid. Yield: 46%; m.p. 97–102 °C; R_f_ = 0.29; ESI HRMS (*m*/*z*): calcd for C_27_H_26_N_5_O_2_^−^ [M − H]^−^ 452.2086, found: 452.2097; ^1^H NMR (501 MHz, acetone-*d*_6_) δ 10.69 (s, 1H), 10.21 (s, 1H), 8.29–8.20 (m, 2H), 7.50–7.43 (m, 2H), 7.41 (dt, *J* = 8.2, 0.9 Hz, 1H), 7.31 (d, *J* = 1.8 Hz, 1H), 7.12 (ddd, *J* = 8.2, 7.1, 1.1 Hz, 1H), 7.08 (dd, *J* = 7.9, 4.7 Hz, 1H), 7.00 (ddd, *J* = 8.0, 7.0, 1.0 Hz, 1H), 6.23 (tt, *J* = 3.7, 1.5 Hz, 1H), 4.39 (ddd, *J* = 9.5, 5.0, 0.8 Hz, 1H), 3.67 (td, *J* = 7.1, 1.3 Hz, 2H), 3.28 (dd, *J* = 17.9, 9.5 Hz, 1H), 3.18–3.12 (m, 2H), 2.82 (dd, *J* = 17.9, 5.1 Hz, 1H), 2.70–2.64 (m, 2H), 2.62–2.55 (m, 2H), 2.52 (t, *J* = 6.8 Hz, 2H), 1.92–1.81 (m, 2H); ^13^C NMR (126 MHz, acetone-*d*_6_) δ 179.0, 177.1, 150.5, 143.9, 138.0, 130.5, 129.2, 127.3, 123.7, 123.2, 122.6, 120.0, 119.8, 119.5, 118.3, 116.6, 112.7, 112.5, 112.5, 56.7, 53.8, 51.2, 38.9, 38.0, 37.2, 29.4, 25.7.

##### Synthesis of 1-(2-(4-(1*H*-Pyrrolo[2,3-*b*]pyridin-3-yl)-3,6-dihydropyridin-1(2*H*)-yl)ethyl)-3-(1*H*-indol-3-yl)pyrrolidine-2,5-dione (**6**)

The title compound was isolated as a yellow solid. Yield: 65%; m.p. 233–235 °C; R_f_ = 0.52; ESI HRMS (*m*/*z*): calcd for C_26_H_25_N_5_O_2_Na^+^ [M + Na]^+^ 462.1906, found: 462.1909; ^1^H NMR (501 MHz, acetone-*d*_6_) δ 10.82 (s, 1H), 10.19 (s, 1H), 8.28–8.24 (m, 2H), 7.56 (d, *J* = 7.8 Hz, 1H), 7.48 (ps, 1H), 7.35 (d, *J* = 8.0 Hz, 1H), 7.33 (d, *J* = 2.4 Hz, 1H), 7.10 (dd, *J* = 7.8, 4.8 Hz, 1H), 7.00 (dt, *J* = 23.3, 7.3 Hz, 2H), 6.24 (d, *J* = 3.7 Hz, 1H), 4.36 (dd, *J* = 9.5, 4.9 Hz, 1H), 3.85–3.67 (m, 2H), 3.29–3.21 (m, 3H), 2.88–2.80 (m, 2H), 2.78–2.67 (m, 3H), 2.57 (td, *J* = 5.9, 2.9 Hz, 2H); ^13^C NMR (126 MHz, acetone-*d*_6_) δ 179.0, 177.1, 150.5, 143.8, 137.9, 130.6, 129.3, 126.9, 124.2, 123.2, 122.5, 120.0, 119.6, 119.6, 118.4, 116.6, 116.6, 112.9, 112.4, 55.4, 54.1, 50.5, 38.9, 37.4, 36.8, 29.2.

##### Synthesis of 1-(4-(4-(1*H*-Pyrrolo[2,3-*b*]pyridin-3-yl)-3,6-dihydropyridin-1(2*H*)-yl)butyl)-3-(5-methoxy-1*H*-indol-3-yl)pyrrolidine-2,5-dione (**7**)

The title compound was isolated as a yellow solid. Yield: 47%; m.p. 140–144 °C; R_f_ = 0.15; ESI HRMS (*m*/*z*): calcd for C_29_H_31_N_5_O_3_Na^+^ [M + Na]^+^ 520.2313, found: 520.2325; ^1^H NMR (400 MHz, CD_3_OD) δ 8.24 (d, *J* = 7.8 Hz, 1H), 8.21 (d, *J* = 4.8 Hz, 1H), 7.47 (d, *J* = 2.9 Hz, 1H), 7.28 (d, *J* = 8.8 Hz, 1H), 7.21 (s, 1H), 7.16 (dd, *J* = 8.0, 4.8 Hz, 1H), 6.94 (d, *J* = 2.4 Hz, 1H), 6.81 (dd, *J* = 8.9, 2.3 Hz, 1H), 6.05 (ps, 1H), 4.38 (dd, *J* = 9.4, 4.3 Hz, 1H), 3.78 (s, 3H), 3.67–3.57 (m, 4H), 3.38–3.17 (m, 3H), 3.16–3.02 (m, 2H), 2.90 (dd, *J* = 18.2, 4.4 Hz, 1H), 2.79–2.71 (m, 2H), 1.78–1.63 (m, 4H); ^13^C NMR (101 MHz, CD_3_OD) δ 181.1, 179.0, 155.3, 149.9, 143.8, 133.7, 131.2, 130.4, 127.6, 124.9, 124.6, 119.1, 117.3, 114.2, 113.5, 113.0, 111.9, 101.7, 56.9, 56.4, 52.1, 50.6, 39.6, 38.6, 37.1, 26.4, 26.0, 25.2, 22.8.

##### Synthesis of 1-(3-(4-(1*H*-Pyrrolo[2,3-*b*]pyridin-3-yl)-3,6-dihydropyridin-1(2*H*)-yl)propyl)-3-(5-methoxy-1*H*-indol-3-yl)pyrrolidine-2,5-dione (**8**)

The title compound was isolated as a yellow solid. Yield: 41%; m.p. 111–115 °C; R_f_ = 0.31; ESI HRMS (*m*/*z*): calcd for C_28_H_29_N_5_O_3_Na^+^ [M + Na]^+^ 506.2155, found: 506.2168; ^1^H NMR (501 MHz, DMSO-*d*_6_) δ 11.60 (d, *J* = 2.7 Hz, 1H), 10.87 (d, *J* = 2.5 Hz, 1H), 8.21–8.16 (m, 2H), 7.47 (d, *J* = 2.6 Hz, 1H), 7.28–7.23 (m, 2H), 7.05 (dd, *J* = 7.8, 4.9 Hz, 1H), 6.82 (d, *J* = 2.4 Hz, 1H), 6.74 (dd, *J* = 8.7, 2.4 Hz, 1H), 6.16–6.11 (m, 1H), 4.30 (dd, *J* = 9.4, 4.9 Hz, 1H), 3.70 (s, 3H), 3.53 (t, *J* = 7.2 Hz, 2H), 3.21 (dd, *J* = 18.0, 9.4 Hz, 1H), 3.06 (p, *J* = 2.4 Hz, 2H), 2.79 (dd, *J* = 18.0, 5.0 Hz, 1H), 2.58 (t, *J* = 5.7 Hz, 2H), 2.41 (t, *J* = 6.9 Hz, 2H), 1.76 (ddt, *J* = 13.7, 10.6, 6.8 Hz, 2H); ^13^C NMR (126 MHz, DMSO-*d*_6_) δ 178.4, 176.7, 153.2, 149.1, 142.6, 131.6, 129.2, 128.3, 126.3, 124.0, 122.9, 118.3, 116.9, 115.6, 114.6, 112.4, 111.4, 110.5, 100.3, 55.3, 55.2, 52.6, 49.9, 37.5, 36.7, 36.0, 28.0, 24.8.

##### Synthesis of 1-(2-(4-(1*H*-Pyrrolo[2,3-*b*]pyridin-3-yl)-3,6-dihydropyridin-1(2*H*)-yl)ethyl)-3-(5-methoxy-1*H*-indol-3-yl)pyrrolidine-2,5-dione (**9**)

The title compound was isolated as a yellow solid. Yield: 43%; m.p. 129–132 °C; R_f_ = 0.53; ESI HRMS (*m*/*z*): calcd for C_27_H_27_N_5_O_3_^+^ [M + H]^+^ 492.2012, found: 492.2000; ^1^H NMR (501 MHz, acetone-*d*_6_) δ 10.64 (s, 1H), 10.01 (s, 1H), 8.28–8.19 (m, 2H), 7.48 (d, *J* = 2.2 Hz, 1H), 7.32–7.29 (m, 1H), 7.27 (dd, *J* = 8.8, 0.6 Hz, 1H), 7.12–7.06 (m, 1H), 7.02 (dd, *J* = 2.5, 0.6 Hz, 1H), 6.80–6.75 (m, 1H), 6.21 (tt, *J* = 3.5, 1.5 Hz, 1H), 4.38 (ddd, *J* = 9.4, 4.7, 0.7 Hz, 1H), 3.79–3.74 (m, 5H), 3.31 (dd, *J* = 18.0, 9.4 Hz, 1H), 3.26 (q, *J* = 2.8 Hz, 2H), 2.83–2.75 (m, 3H), 2.71 (t, *J* = 6.6 Hz, 2H), 2.59–2.53 (m, 2H); ^13^C NMR (126 MHz, acetone-*d*_6_) δ 178.9, 177.0, 155.0, 150.5, 143.9, 133.0, 130.6, 129.2, 127.7, 124.3, 123.1, 119.6, 118.3, 116.6, 113.1, 113.1, 112.8, 112.7, 101.5, 56.0, 55.7, 54.0, 50.7, 38.8, 37.3, 36.9, 29.2.

##### Synthesis of 1-(4-(4-(1*H*-Pyrrolo[2,3-*b*]pyridin-3-yl)-3,6-dihydropyridin-1(2*H*)-yl)butyl)-3-(5-fluoro-1*H*-indol-3-yl)pyrrolidine-2,5-dione (**10**)

The title compound was isolated as a yellow solid. Yield: 49%; m.p. 98–102 °C; R_f_ = 0.12; ESI HRMS (*m*/*z*): calcd for C_28_H_28_N_5_O_2_F^+^ [M + H]^+^ 486.2325, found: 485.3199; ^1^H NMR (501 MHz, acetone-*d*_6_) δ 10.67 (bs, 1H), 10.37 (bs, 1H), 8.25–8.20 (m, 2H), 7.48 (d, *J* = 2.0 Hz, 1H), 7.46–7.39 (m, 2H), 7.27 (ddt, *J* = 9.9, 2.5, 0.7 Hz, 1H), 7.09 (dd, *J* = 7.7, 5.0 Hz, 1H), 6.97–6.90 (m, 1H), 6.18 (td, *J* = 3.5, 1.8 Hz, 1H), 4.43 (ddd, *J* = 9.4, 5.0, 0.8 Hz, 1H), 3.58 (t, *J* = 7.0 Hz, 2H), 3.33 (dd, *J* = 18.0, 9.5 Hz, 1H), 3.19 (s, 2H), 2.87 (dd, *J* = 18.0, 5.0 Hz, 1H), 2.73 (d, *J* = 5.9 Hz, 2H), 2.60 (s, 2H), 2.54 (t, *J* = 6.9 Hz, 2H), 1.74–1.65 (m, 2H), 1.60 (p, *J* = 7.2 Hz, 2H); ^13^C NMR (101 MHz, DMSO-*d*_6_) δ 178.3, 176.6, 156.8 (d, *J* = 231.6 Hz), 149.1, 142.8, 133.1, 129.3, 128.3, 126.3 (d, *J* = 10.0 Hz), 125.4, 123.5, 116.8, 115.8, 114.0, 113.8, 112.7 (d, *J* = 9.7 Hz), 111.0 (d, *J* = 4.7 Hz), 109.6 (d, *J* = 26.0 Hz), 103.34 (d, *J* = 23.4 Hz), 62.0, 56.4, 51.6, 49.4, 37.9, 37.4, 35.6, 25.5, 25.0.

##### Synthesis of 1-(3-(4-(1*H*-Pyrrolo[2,3-*b*]pyridin-3-yl)-3,6-dihydropyridin-1(2*H*)-yl)propyl)-3-(5-fluoro-1*H*-indol-3-yl)pyrrolidine-2,5-dione (**11**)

The title compound was isolated as a yellow solid. Yield: 53%; m.p. 147–150 °C; R_f_ = 0.26; ESI HRMS (*m*/*z*): calcd for C_27_H_25_FN_5_O_2_^−^ [M − H]^−^ 470.1992, found: 470.2012; ^1^H NMR (501 MHz, acetone-*d*_6_) δ 10.65 (bs, 1H), 10.33 (bs, 1H), 8.26–8.19 (m, 2H), 7.48 (d, *J* = 2.1 Hz, 1H), 7.42–7.38 (m, 2H), 7.22 (ddt, *J* = 9.9, 2.5, 0.7 Hz, 1H), 7.08 (dd, *J* = 7.9, 4.8 Hz, 1H), 6.95–6.89 (m, 1H), 6.22 (dq, *J* = 3.6, 1.8 Hz, 1H), 4.38 (ddd, *J* = 9.5, 5.2, 0.8 Hz, 1H), 3.66 (td, *J* = 7.1, 0.9 Hz, 2H), 3.29 (dd, *J* = 17.9, 9.5 Hz, 1H), 3.15 (q, *J* = 2.8 Hz, 2H), 2.84 (dd, *J* = 17.9, 5.2 Hz, 1H), 2.68–2.64 (m, 2H), 2.61–2.55 (m, 2H), 2.51 (t, *J* = 6.8 Hz, 2H), 1.92–1.80 (m, 2H); ^13^C NMR (126 MHz, acetone-*d*_6_) δ 178.8, 176.9, 158.4 (d, *J* = 232.5 Hz), 150.5, 143.9, 134.5, 130.5, 129.2, 127.7 (d, *J* = 9.9 Hz), 125.6, 123.1, 119.8, 118.3, 116.6, 113.4 (d, *J* = 9.7 Hz), 113.4, 112.9 (d, *J* = 4.7 Hz), 110.7 (d, *J* = 26.4 Hz), 104.4 (d, *J* = 23.7 Hz), 56.7, 53.8, 51.2, 38.7, 38.0, 36.9, 29.4, 25.7.

##### Synthesis of 1-(2-(4-(1*H*-Pyrrolo[2,3-*b*]pyridin-3-yl)-3,6-dihydropyridin-1(2*H*)-yl)ethyl)-3-(5-fluoro-1*H*-indol-3-yl)pyrrolidine-2,5-dione (**12**)

The title compound was isolated as a yellow solid. Yield: 51%; m.p. 250–252 °C; R_f_ = 0.53; ESI HRMS (*m*/*z*): calcd for C_26_H_24_N_5_O_2_FNa^+^ [M + Na]^+^ 480.1812, found: 480.1812; ^1^H NMR (500 MHz, DMSO-*d*_6_) δ 11.62 (d, *J* = 2.7 Hz, 1H), 11.14 (d, *J* = 2.7 Hz, 1H), 8.23–8.17 (m, 2H), 7.48 (d, *J* = 2.6 Hz, 1H), 7.40 (d, *J* = 2.5 Hz, 1H), 7.35 (dd, *J* = 8.8, 4.5 Hz, 1H), 7.18 (dd, *J* = 9.9, 2.6 Hz, 1H), 7.08 (dd, *J* = 7.9, 4.7 Hz, 1H), 6.92 (td, *J* = 9.2, 2.5 Hz, 1H), 6.13 (d, *J* = 3.7 Hz, 1H), 4.36 (dd, *J* = 9.3, 5.1 Hz, 1H), 3.74–3.59 (m, 2H), 3.27–3.13 (m, 3H), 2.78 (dd, *J* = 18.1, 5.1 Hz, 1H), 2.76–2.55 (m, 4H), 2.47 (d, *J* = 6.4 Hz, 2H); ^13^C NMR (126 MHz, DMSO-*d*_6_) δ 178.2, 176.4, 156.8 (d, *J* = 231.8 Hz), 149.1, 142.6, 133.1, 129.2, 128.3, 126.0 (d, *J* = 10.0 Hz), 125.6, 122.9, 118.1, 116.9, 115.6, 114.7, 112.7 (d, *J* = 9.8 Hz), 111.2 (d, *J* = 4.7 Hz), 109.5 (d, *J* = 26.2 Hz), 103.2 (d, *J* = 23.3 Hz), 54.5, 52.7, 49.6, 39.0, 37.4, 35.9, 27.7.

### 3.2. Binding Assays

#### 3.2.1. Methodology of Radioligand Binding Assay

Preparation of solutions of test and reference compounds.

One-millimolar stock solutions of tested compounds were prepared in DMSO. Serial dilutions of compounds were prepared in a 96-well microplate using assay buffers with the automated pipetting system epMotion 5070 (Eppendorf, Hamburg, Germany). Each compound was tested in 6 concentrations from 10–5 to 10–10 M (final concentration).

##### Serotonin Transporter Binding Assay

Radioligand binding was performed using membranes from HEK-293 cells stably transfected with the human serotonin transporter (PerkinElmer, Waltham, MA, USA). All assays were carried out in duplicates. Working solutions of 50 µL of the tested compounds, 50 µL of [^3^H]-imipramine (final concentration 2 nM) and 150 µL of diluted membranes (9 µg of protein per well) prepared in assay buffer (50 mM Tris, pH 7.4, 5 mM KCl, 120 mM NaCl) were transferred to a polypropylene 96-well microplate using the 96-well pipetting station Rainin Liquidator (MettlerToledo, Beverly, MA, USA). Imipramine (10 μM) was used to define nonspecific binding. The microplate was covered with sealing tape, mixed and incubated for 30 min at 27 °C. The reaction was terminated by rapid filtration through a GF/C filter mate pre-soaked with 0.5% polyethyleneimine for 30 min. Ten rapid washes with 200 µL of 50 mM Tris buffer and 154 mM NaCl (4 °C, pH 7.4) were performed using the automated harvester system Harvester-96 MACH III FM (Tomtec, Hamden, CT, USA). The filter mates were dried at 37 °C in a forced-air fan incubator, and then solid scintillator MeltiLex was melted on filter mates at 90 °C for 4 min. Radioactivity was counted in a MicroBeta2 scintillation counter (PerkinElmer, Turku, Finland). Data were fitted to a one-site curve-fitting equation with Prism 6 (GraphPad Software), and Ki values were estimated using the Cheng–Prusoff equation.

##### 5-HT_1A_ Receptor Binding Assay

Radioligand binding was performed using membranes from CHO-K1 cells stably transfected with the human 5-HT_1A_ receptor (PerkinElmer, Waltham, MA, USA). All assays were carried out in duplicates. A measure of 50 µL of working solution of the tested compounds, 50 µL of [^3^H]-8-OH-DPAT (final concentration: 1 nM) and 150 µL of diluted membranes (10 µg of protein per well) prepared in assay buffer (50 mM Tris, pH 7.4, 10 mM MgSO_4_, 0.5 mM EDTA, 0.1% ascorbic acid) were transferred to a polypropylene 96-well microplate using the 96-well pipetting station Rainin Liquidator (MettlerToledo, Beverly, MA, USA). Serotonin (10 μM) was used to define nonspecific binding. The microplate was covered with sealing tape, mixed and incubated for 60 min at 27 °C. The reaction was terminated by rapid filtration through GF/C filter mate pre-soaked with 0.3% polyethyleneimine for 30 min. Ten rapid washes with 200 µL of 50 mM Tris buffer (4 °C, pH 7.4) were performed using the automated harvester system Harvester-96 MACH III FM (Tomtec, Hamden, CT, USA). The filter mates were dried at 37 °C in a forced-air fan incubator, and then solid scintillator MeltiLex was melted on filter mates at 90 °C for 4 min. Radioactivity was counted using a MicroBeta2 scintillation counter (PerkinElmer, Turku, Finland). Data were fitted to a one-site curve-fitting equation with Prism 6 (GraphPad Software), and *K*_i_ values were estimated using the Cheng–Prusoff equation.

##### D_2_ Receptor Binding Assay

Radioligand binding was performed using membranes from CHO-K1 cells stably transfected with the human D_2_ receptor (LifeTechnologies, Waltham, MA, USA). All assays were carried out in duplicates. Measure of 50 µL of working solution of the tested compounds, 50 µL of [^3^H]-methylspiperone (final concentration 1 nM) and 150 µL of diluted membranes (5 µg protein per well) prepared in assay buffer (50 mM HEPES, pH 7.4, 50 mM NaCl, 5 mM MgCl_2_, 0.5 mM EDTA) were transferred to a 96-well microplate. Haloperidol (10 μM) was used to define nonspecific binding. The microplate was covered with sealing tape, mixed and incubated for 60 min at 37 °C. The reaction was terminated by rapid filtration through GF/C filter mate pre-soaked with 0.5% polyethyleneimine for 30 min. Ten rapid washes with 200 µL of 50 mM Tris buffer (4 °C, pH 7.4) were performed using the automated harvester system Harvester-96 MACH III FM (Tomtec, Hamden, CT, USA). The filter mates were dried at 37 °C in a forced-air fan incubator, and then solid scintillator MeltiLex was melted on filter mates at 90 °C for 4 min. Radioactivity was counted using a MicroBeta2 scintillation counter (PerkinElmer, Turku, Finland). Data were fitted to a one-site curve-fitting equation with Prism 6 (GraphPad Software), and *K*_i_ values were estimated using the Cheng–Prusoff equation.

##### ADRA1 Receptor Binding Assay

Radioligand binding was performed using tissue rat cortex. All assays were carried out in duplicates. Measures of 50 µL of working solution of the tested compounds, 50 µL of [^3^H]-prazosin (final concentration 0.2 nM) and 150 µL of tissue suspension prepared in assay buffer (50 mM Tris-HCl, pH 7.6) were transferred to a polypropylene 96-well microplate using the 96-well pipetting station Rainin Liquidator (MettlerToledo). Phentolamine (10 μM) was used to define nonspecific binding. The microplate was covered with sealing tape, mixed and incubated for 30 min at 30 °C. The reaction was terminated by rapid filtration through GF/B filter mate. Ten rapid washes with 200 µL of 50 mM Tris buffer (4 °C, pH 7.6) were performed using a 96-well FilterMate harvester (PerkinElmer, Waltham, MA, USA ). The filter mates were dried at 37 °C in a forced-air fan incubator, and then solid scintillator MeltiLex was melted on filter mates at 90 °C for 4 min. The radioactivity on the filter was measured using a MicroBeta TriLux 1450 scintillation counter (PerkinElmer, Waltham, MA, USA). Data were fitted to a one-site curve-fitting equation with Prism 6 (GraphPad Software), and *K*_i_ values were estimated using the Cheng–Prusoff equation.

##### 5-HT_2A_ Binding Assay

Tissue preparations (rat cortex) and competition binding experiments for 5-HT_2A_ receptors were carried out according to a previously described, standard technique using 0.5 nM of [^3^H]-ketanserin as the radioligand and 10 μM of methysergide to determine non-specific binding. Following 20 min of incubation, the samples containing receptor homogenates and the investigated compounds (7–9 concentrations run in triplicate) were rapidly filtered under vacuum through GF/B glass fibre filters; the filters were washed extensively with an ice-cold buffer using a Brandel harvester. Bound radioactivity was measured by scintillation counting using a liquid scintillation cocktail. The inhibition constants (*K*_i_) were calculated using the Cheng–Prusoff equation. Results are expressed as the means of at least three separate experiments. 

##### Receptor Binding Experiments with HEK293 Cells Expressing Human 5-HT_6_ and 5-HT_7_ Receptors

Cell culture and preparation of cell membranes.

HEK293 cells with stable expression of human serotonin 5-HT_6_ and 5-HT_7b_ receptors (prepared with the use of Lipofectamine 2000(Carlsbad, CA, USA)) were maintained at 37 °C in a humidified atmosphere with 5% CO_2_ and grown in Dulbecco’s Modifier Eagle Medium containing 10% dialyzed foetal bovine serum and 500 μg/mL G418 sulphate. For membrane preparation, cells were subcultured in 10 cm-diameter dishes, grown to 90% confluence, washed twice with prewarmed to 37 °C phosphate buffered saline (PBS) and pelleted by centrifugation (200 g) in PBS containing 0.1 mM EDTA and 1 mM dithiothreitol. Prior to membrane preparation, pellets were stored at −80 °C.

Radioligand binding assays.

Cell pellets were thawed and homogenised in 20 volumes of assay buffer using an Ultra Turrax tissue homogeniser and centrifuged twice at 35.00 g for 20 min at 4 °C, with incubation for 15 min at 37 °C in between. The composition of the assay buffers was as follows: for 5-HT_6_R, 50 mM Tris-HCl, 0.5 mM EDTA and 4 mM MgCl_2_; and for 5-HT_7b_R, 50 mM Tris-HCl, 4 mM MgCl_2_, 10 μM pargyline and 0.1% ascorbate. All assays were incubated in a total volume of 200 μL in 96-well microtitre plates for 1 h at 37 °C. The process of equilibration was terminated by rapid filtration through Unifilter plates with a 96-well cell harvester, and radioactivity retained on the filters was quantified on a Microbeta plate reader. For displacement studies, the assay samples contained radioligands such as 2 nM [^3^H]-LSD (85.2 Ci/mmol for 5-HT_6_R) or 0.6 nM [^3^H]-5-CT (39.2 Ci/mmol) for 5-HT_7_R. Non-specific binding was defined using 10 μM methiothepine in the 5-HT_6_R or 5-HT_7_R binding experiments, respectively. Each compound was tested in triplicate at 7–8 concentrations (10^−11^–10^−4^ M). The inhibition constants (*K*_i_) were calculated using the Cheng–Prusoff equation. Membrane preparation and general assay procedures for cloned receptors were adjusted to a 96-microwell format based on protocols previously described by us [38].

#### 3.2.2. Methodology of DAT and NET Radioligand Binding Assay

Preparation of solutions of test and reference compounds.

Ten-micromolar stock solutions of tested compounds were prepared in DMSO. Serial dilutions of compounds were prepared in a 96-well microplate in assay buffers using the automated pipetting system epMotion 5070 (Eppendorf, Hamburg, Germany). Each compound was tested in a screening assay at final concentrations of 10 µM and 1 µM. Results were expressed as percent inhibition of radioligand binding. Reference compounds were tested in 8 concentrations from 10^−5^ to 10^−12^ M (final concentration).

##### DAT Binding Assay

Radioligand binding was performed using membranes from CHO-K1 cells stably transfected with the human dopamine transporter (PerkinElmer, Waltham, MA, USA). All assays were carried out in duplicates. Measures of 50 µL of working solution of the tested compounds, 50 µL of [^3^H]-WIN 35,428 (final concentration 25 nM) and 150 µL of diluted membranes (12 µg protein per well) prepared in assay buffer (50 mM Tris-HCl, pH 7.4, 100 mM NaCl) were transferred to a polypropylene 96-well microplate using the 96-well pipetting station Rainin Liquidator (MettlerToledo, Beverly, MA, USA) [39]. GBR 12909 (10 μM) was used to define nonspecific binding. The microplate was covered with sealing tape, mixed and incubated for 120 min at 4 °C. The reaction was terminated by rapid filtration through GF/C filter mate pre-soaked with 0.5% polyethyleneimine for 30 min. Ten rapid washes with 200 µL of 50 mM Tris buffer (4 °C, pH 7.4) were performed using the automated harvester system Harvester-96 MACH III FM (Tomtec, Hamden, CT, USA). The filter mates were dried at 37 °C in a forced-air fan incubator, and then solid scintillator MeltiLex was melted on filter mates at 90 °C for 4 min. Radioactivity was counted using a MicroBeta2 scintillation counter (PerkinElmer, Turku, Finland). Data were fitted to a one-site curve-fitting equation with Prism 5 (GraphPad Software), and *K*_i_ values were estimated using the Cheng–Prusoff equation.

##### NET Binding Assay

Radioligand binding was performed using membranes from MDCK cells stably transfected with the human norepinephrine transporter (PerkinElmer, Waltham, MA, USA). All assays were carried out in duplicates. Measures of 50 µL of working solution of the tested compounds, 50 µL of [^3^H]-nisoxetine (final concentration 4 nM) and 150 µL of diluted membranes (3 µg protein per well) prepared in assay buffer (50 mM Tris-HCl, pH 7.4, 120 mM NaCl; 5 mM KCl) were transferred to a polypropylene 96-well microplate using the 96-well pipetting station Rainin Liquidator (MettlerToledo, Beverly, MA, USA). Desipramine (1 μM) was used to define nonspecific binding. The microplate was covered with sealing tape, mixed and incubated for 60 min at 4 °C. The reaction was terminated by rapid filtration through GF/C filter mate pre-soaked with 0.5% polyethyleneimine for 30 min. Ten rapid washes with 200 µL of 50 mM Tris buffer (4 °C, pH 7.4) were performed using the automated harvester system Harvester-96 MACH III FM (Tomtec, Hamden, CT, USA). The filter mates were dried at 37 °C in a forced-air fan incubator, and then solid scintillator MeltiLex was melted on filter mates at 90 °C for 4 min. Radioactivity was counted using a MicroBeta2 scintillation counter (PerkinElmer, Turku, Finland). Data were fitted to a one-site curve-fitting equation with Prism 5 (GraphPad Software), and *K*_i_ values were estimated using the Cheng–Prusoff equation.

## 4. Conclusions

To develop multi-target 3-(1*H*-indol-3-yl)pyrrolidine-2,5-diones derivatives with dual 5-HT_1A_ receptor and SERT binding, we synthesised 1-(4-(7-azaindole)-3,6-dihydropyridin-1-yl)alkyl-3-(1*H*-indol-3-yl)pyrrolidine-2,5-dione derivatives **4**–**12**. Based on the receptor binding assays we have conducted so far, some general conclusions can be drawn. First, replacement of the 3-(1,2,3,6-tetrahydropyridin-4-ly)-1*H*-indole moiety with 3-(1,2,3,6-tetrahydropyridin-4-yl)-1*H*-pyrrolo[2,3-*b*]pyridine resulted in the loss of affinity for the 5-HT_1A_ receptor; however, compounds **4**–**12** displayed good affinity for SERT. The compounds with the highest affinity for SERT were compound **4** (*K*_i_ = 47.0 nM), compound **8** (*K*_i_ = 23.0 nM), and compound **11** (*K*_i_ = 9.2 nM). Compound **11** was the only one to display dual-binding affinity for the 5-HT_1A_ receptor and SERT (*K*_i_ = 128.0 and 9.2 nM, respectively). Compounds **4**, **8**, and **11** were D_2_ receptor ligands, with compound **8** (R = OCH_3_) presenting the highest affinity (*K*_i_ = 13 nM). Replacing 1*H*-indole with 7-azaindole did not significantly affect the affinity for the D_2_ receptor. These findings suggest that the 3-(1*H*-indol-3-yl)pyrrolidine-2,5-dione part of the molecule plays a crucial role in the interaction with the D_2_ receptor binding site. Considering compounds **4**, **8**, and **11** as the most promising because of their convenient receptor profiles, we selected them for a follow-up evaluation of their affinity for DAT and NET. The assays revealed that compounds **4** and **11** exhibited the most desired binding profile to SERT/NET/DAT among the series: *K*_i_ = 47.0 nM/167.0 nM/43% and *K*_i_ = 9.2 nM/40%/288.0 nM, respectively. In summary, compounds **4** and **11** represent templates for the future development of multi-target ADDs, given their favourable binding profiles across the key MATs and monoamine receptors. In a follow-up study to validate the potential of compounds **4** and **11**, functional activity should be evaluated through in vivo studies using animal models. These studies will help verify the antidepressant activity of the obtained compounds and assess their potential as future antidepressants.

## Figures and Tables

**Table 1:**
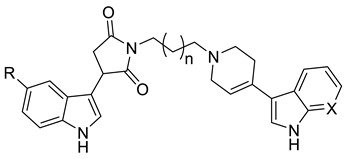
SERT and the 5-HT_1A_ receptor binding affinities of 3-(1,2,3,6-tetrahydropyridin-4-yl)-1*H*-pyrrolo[2,3-*b*]pyridine derivatives **4**–**12** and their 3-(1,2,3,6-tetrahydropyridin-4-ly)-1*H*-indole analogues **13**–**21**.

Compound	R	X	*n*	*K*_i_ ± SEM [nM] ^a^	
				5-HT_1A_	SERT
4	H	N	2	660.0 ± 60.2	47.0 ± 5.0
5	H	N	1	2450.0 ± 24.0	57.1 ± 3.2
6	H	N	0	NC	245 ± 18
7	OCH_3_	N	2	824.4 ± 23.2	58.4 ± 4.0
8	OCH_3_	N	1	NC	23.0 ± 2.8
9	OCH_3_	N	0	NC	92.0 ± 8.0
10	F	N	2	128.0 ± 12.0	77.6 ± 2.5
11	F	N	1	NC	9.2 ± 0.6
12	F	N	0	NC	252.0 ± 24.0
13 [19]	H	CH	2	12.5 ± 1.7	11.3 ± 0.6
14 [19]	H	CH	1	161.4 ± 17.5	4.0 ± 0.1
15 [19]	H	CH	0	249.5 ± 6.8	33.8 ± 3.2
16 [21]	OCH_3_	CH	2	30.4 ± 1.8	2.6 ± 0.3
17 [21]	OCH_3_	CH	1	221.0 ± 11.5	782.0 ± 54.0
18 [21]	OCH_3_	CH	0	347.7 ± 12.1	14.7 ± 1.5
19 [21]	F	CH	2	48.2 ± 2.9	33.3 ± 2.9
20 [21]	F	CH	1	190.0 ± 8.5	1.5 ± 0.2
21 [21]	F	CH	0	NT	NT
serotonin				1.5 ± 0.1	NT
imipramine				NT	30.0 ± 2.7

^a^—The inhibition constants (*K*_i_) were calculated using the Cheng–Prushoff equation. Data expressed as the mean ± SEM of three independent experiments carried out in duplicate. NC—not converged; NT—not tested.

**Table 2:**
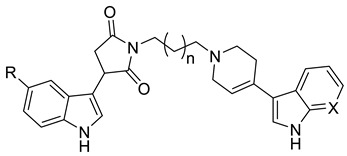
Structures and in vitro affinities of the synthesised compounds **4**, **8**, **11** and **20** (indole-based analogue of compound **11**) to the 5-HT_2A_, 5-HT_6_, 5-HT_7_ and D_2_ receptors.

Compound	R	X	*n*	*K*_i_ ± SEM [nM] ^a^			
				5-HT_2A_	5-HT_6_	5-TH_7_	D_2_
4	H	N	2	1398 ± 71	1691 ± 186	1416 ± 172	78 ± 7
8	OCH_3_	N	1	727 ± 43	2973 ± 138	1533 ± 76	13 ± 2
11	F	N	1	1158 ± 96	1293 ± 147	1126 ± 89	51 ± 6
20	F	CH	1	237 ± 17	257 ± 17	30 ± 4	56.0 ± 7.0

^a^—The inhibition constants (*K*_i_) were calculated using the Cheng–Prushoff equation. Results are expressed as means ± SEM of at least three separate experiments.

**Table 3:** The α_1_-adrenoceptor binding affinities of compounds **4**, **6**, **8**, **9** and **12**.

Compound	R	X	*n*	*K*_i_ ± SEM [nM] ^a^
				α_1_
4	H	N	2	87.0 ± 2.0
6	H	N	0	367.0 ± 55.2
8	OCH_3_	N	1	265.0 ± 6.5
9	OCH_3_	N	0	319.0 ± 10.2
12	F	N	0	456.0 ± 6.0
phentolamine				11.0 ± 0.7

^a^—The inhibition constants (*K*_i_) were calculated using the Cheng–Prushoff equation. Results are expressed as means ± SEM of at least three separate experiments.

**Table 4:** MATs binding affinities of selected 3-(1,2,3,6-tetrahydropyridin-4-yl)-1*H*-pyrrolo[2,3-*b*]pyridine derivatives.

Compound	Binding Affinity (*K*_i_, nM) ^b^			Selectivity Ratio	
	hSERT	hNET	hDAT	hNET/hSERT	hDAT/hSERT
4	47.0 ± 5.0	167.0 ± 13.8	43% ^a^	3.5	-
6	245.0 ± 18.0	37% ^a^	916.0 ± 40.5	-	3.7
8	23.0 ± 2.8	43% ^a^	663.0 ± 15.0	-	28.8
9	92.0 ± 8.0	41% ^a^	335.0 ± 14.8	-	3.6
11	9.2 ± 0.6	40% ^a^	288.0 ± 25.2	-	31.3
12	252.0 ± 24.0	42% ^a^	229.0 ± 21.4	-	0.9
desipramine	-	3.7 ± 0.1	-	-	-
GBR12909	-	-	4.0 ± 0.2	-	-
imipramine	30.0 ± 2.7	-	-	-	-

^a^—Screening procedure—displacement % at 1 µM; ^b^—all assays were carried out in duplicates. Each compound was tested in a screening assay at a final concentration of 10 µM and 1 µM. Data were fitted to a one-site curve-fitting equation with Prism 5 (GraphPad Software 8.0.1), and *K*_i_ values were estimated using the Cheng–Prusoff equation.

## Data Availability

All data relevant to the publication are included.

## Supplementary Materials

Supplementary data to this article can be found online at https://www.mdpi.com/article/10.3390/ijms252011276/s1.
