# Supplementary material for: Design and Synthesis of Potential Multi-Target Antidepressants: Exploration of 1-(4-(7-Azaindole)-3,6-dihydropyridin-1-yl)alkyl-3-(1H-indol-3-yl)pyrrolidine-2,5-dione Derivatives with Affinity for the Serotonin Transporter
Source: Int J Mol Sci. 2024 Oct 20;25(20):11276. doi: 10.3390/ijms252011276 (PMC11508649; doi:10.3390/ijms252011276)

supplementary materials

Design and synthesis of multi-target antidepressants: exploration of 1-(4-(7-azaindole)-3,6-dihydropyridin-1-yl)alkyl-3-(1*H*-indol-3-yl)pyrrolidine-2,5-dione derivatives with affinity for the serotonin transporter

Martyna Z. Wróbel[a*], Andrzej Chodkowski[a], Agata Siwek[b], Grzegorz Satała[c], Andrzej J. Bojarski[c], Maciej Dawidowski[a]

[a]Department of Drug Technology and Pharmaceutical Biotechnology, Faculty of Pharmacy, Medical University of Warsaw, 1 Banacha Street, 02-097 Warszawa, Poland

[b]Department of Pharmacobiology, Faculty of Pharmacy, Jagiellonian University Medical College, 9 Medyczna Street, 30-688 Kraków, Poland

[c]Department of Medicinal Chemistry, Maj Institute of Pharmacology, Polish Academy of Sciences, 12 Smetna Street, 31-343 Kraków, Poland

* corresponding author, e-mail: martyna.wrobel@wum.edu.pl

1. ^{1}H, ^{13}C and COSY NMR spectra of 1-(4-(4-(1*H*-pyrrolo[2,3-*b*]pyridin-3-yl)-3,6-dihydropyridin-1(2*H*)-yl)butyl)-3-(1*H*-indol-3-yl)pyrrolidine-2,5-dione (**4**)

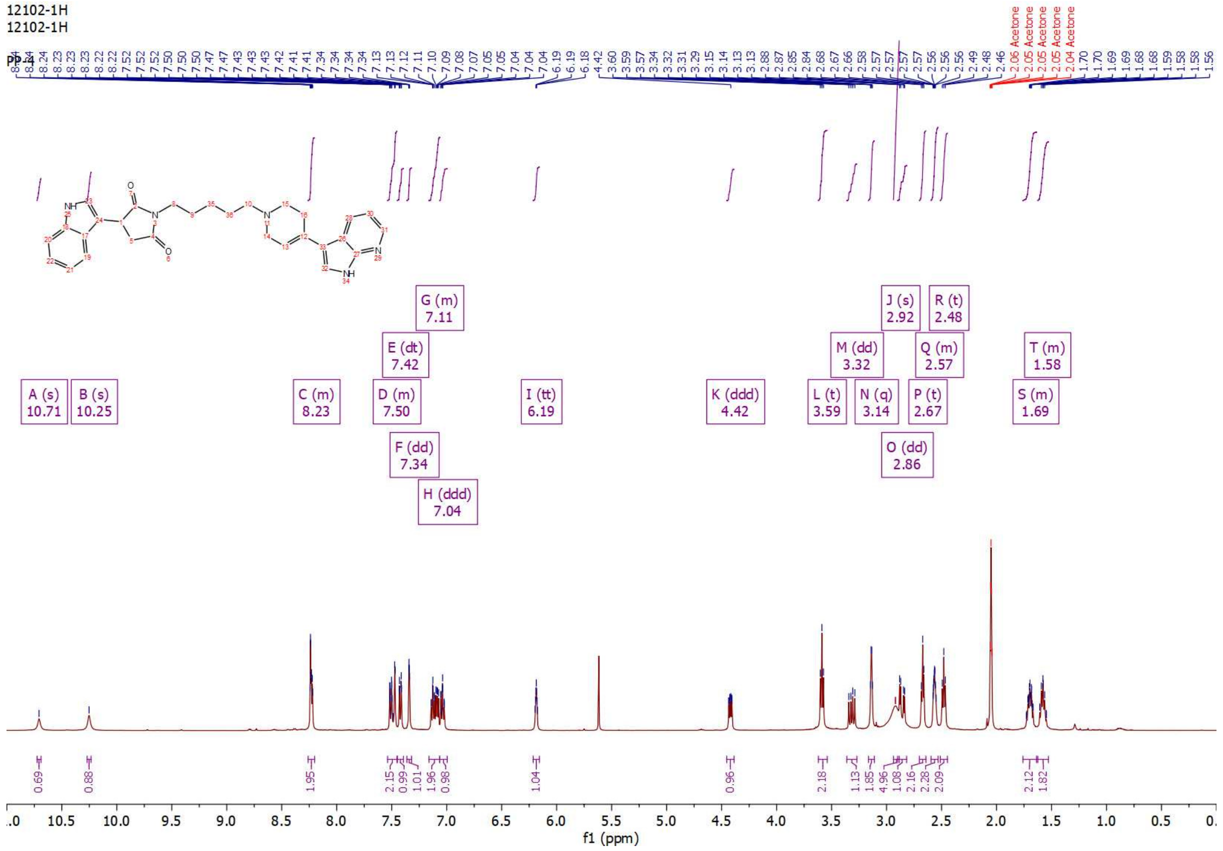

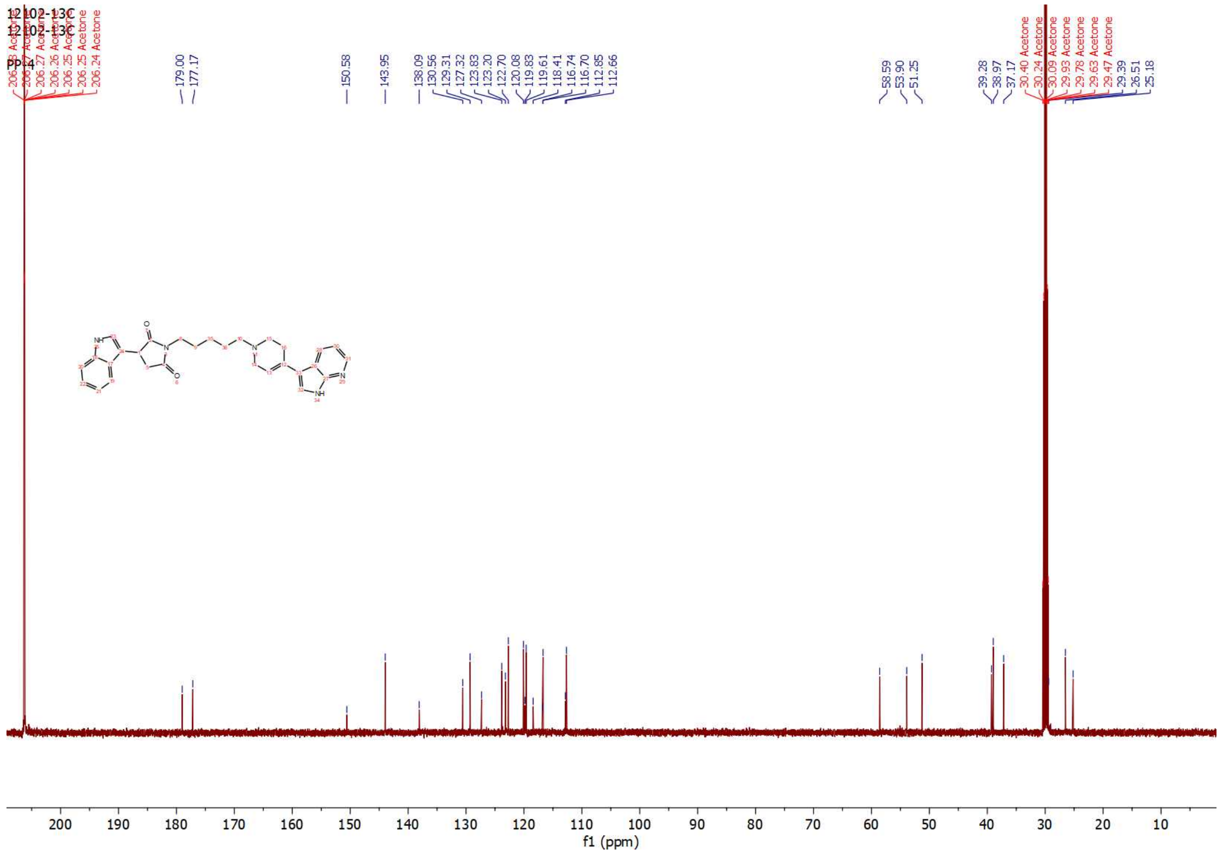
12102-13C
PP-4
179.00
177.17
150.58
143.95
138.09
130.56
129.31
127.32
123.83
123.20
122.70
120.08
119.83
119.61
118.41
116.74
116.70
112.85
112.66
58.59
53.90
51.25
39.28
38.97
37.17
29.39
26.51
25.18
f1 (ppm)

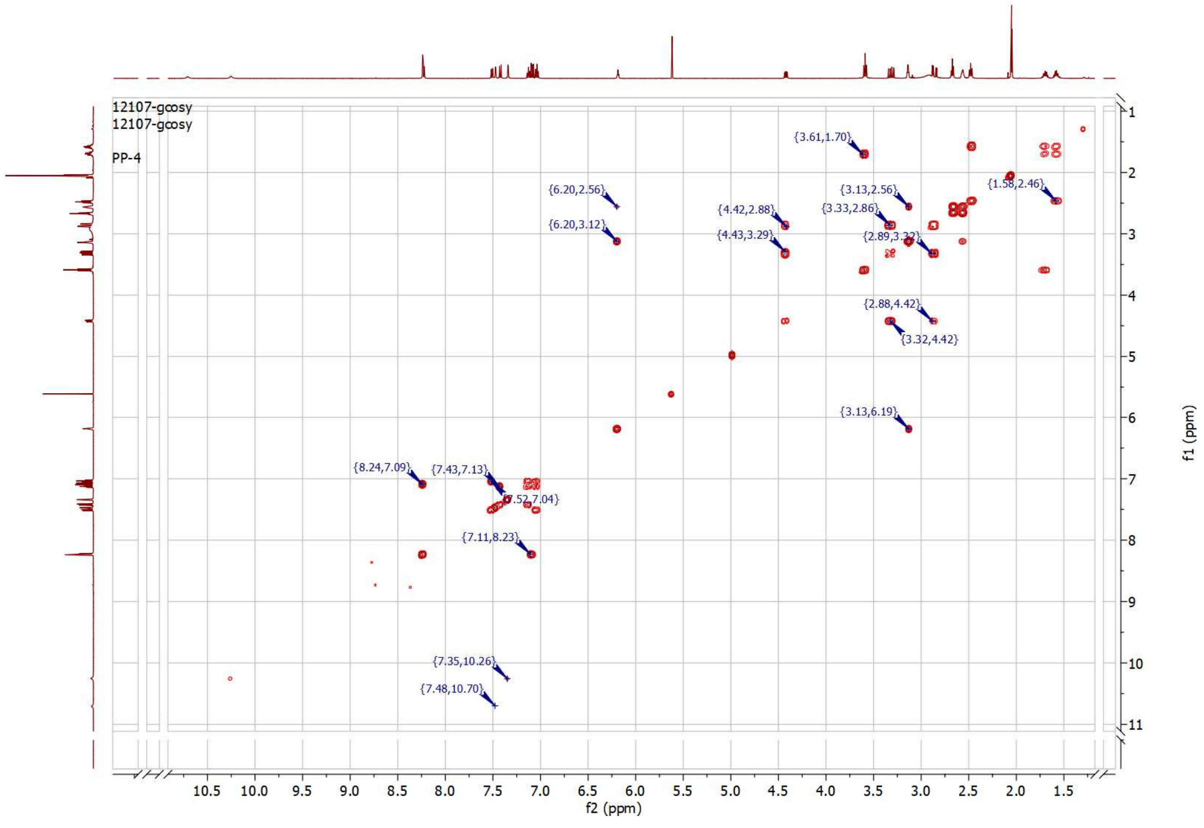
12107-gcosy
PP-4
f2 (ppm)
f1 (ppm)

2. HRMS spectra of 1-(4-(4-(1*H*-pyrrolo[2,3-*b*]pyridin-3-yl)-3,6-dihydropyridin-1(2*H*)-yl)butyl)-3-(1*H*-indol-3-yl)pyrrolidine-2,5-dione (**4**)

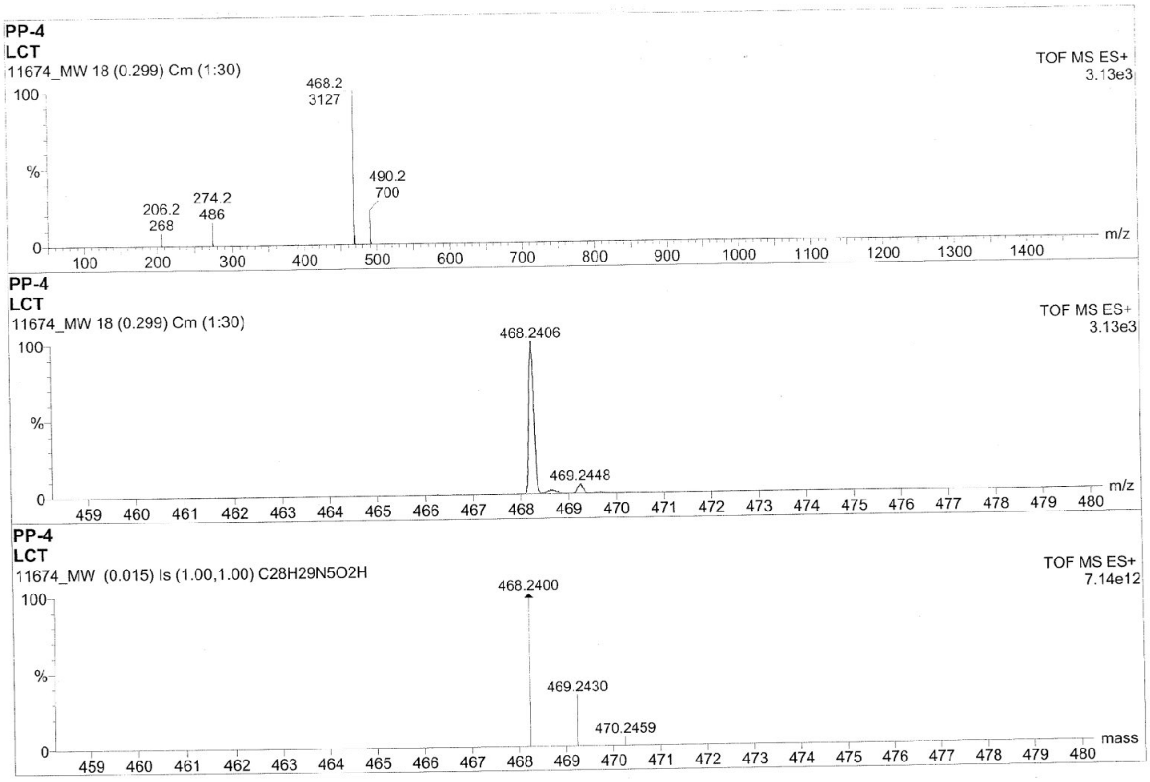

3. ^{1}H and ^{13}C NMR spectra of 1-(3-(4-(1*H*-pyrrolo[2,3-*b*]pyridin-3-yl)-3,6-dihydropyridin-1(2*H*)-yl)propyl)-3-(1*H*-indol-3-yl)pyrrolidine-2,5-dione (**5**)

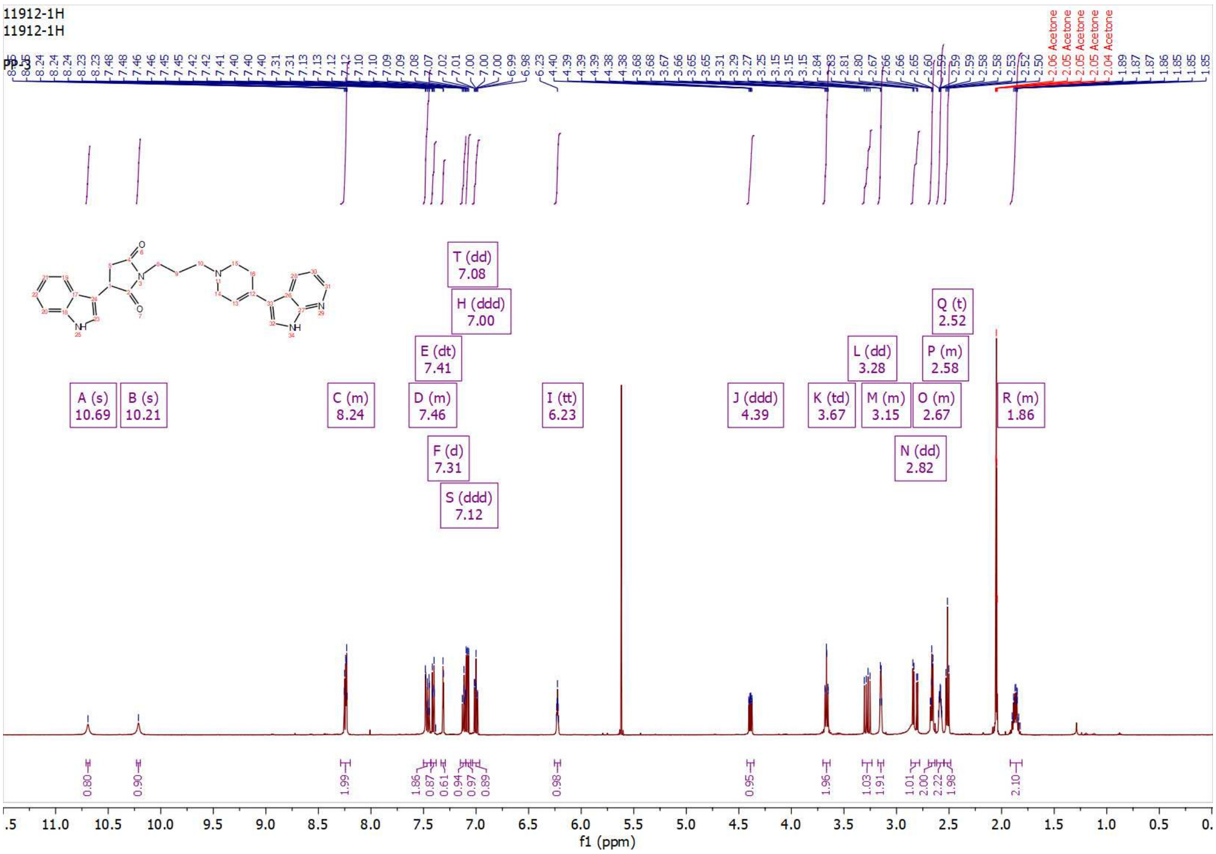

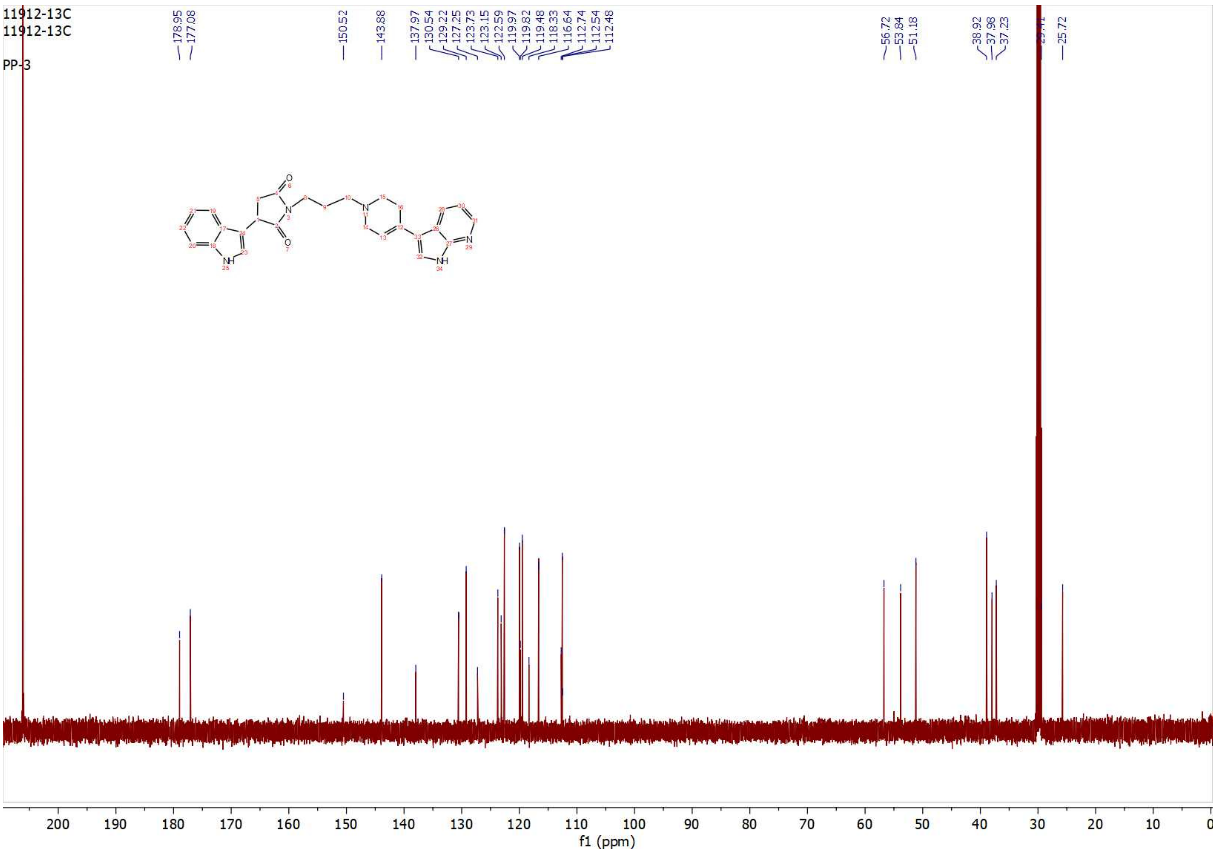

4. HRMS spectra of 1-(3-(4-(1*H*-pyrrolo[2,3-*b*]pyridin-3-yl)-3,6-dihydropyridin-1(2*H*)-yl)propyl)-3-(1*H*-indol-3-yl)pyrrolidine-2,5-dione (**5**)

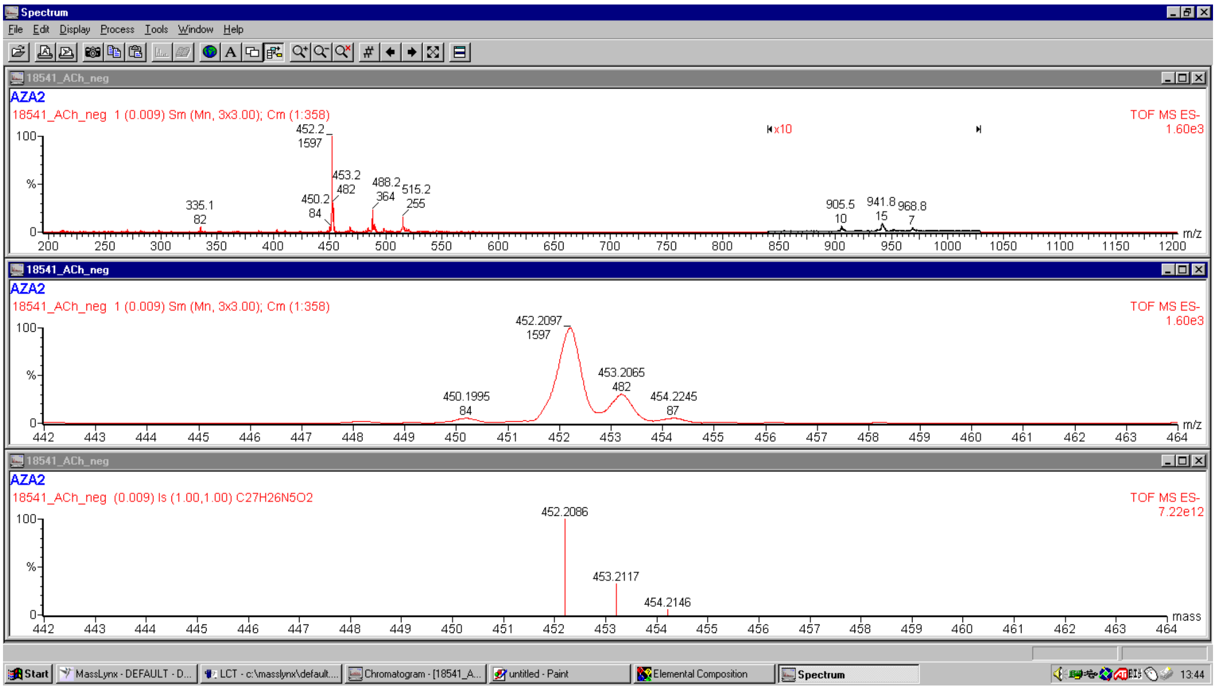

5. ^{1}H, ^{13}C, COSY and HSQC NMR spectra of 1-(2-(4-(1*H*-pyrrolo[2,3-*b*]pyridin-3-yl)-3,6-dihydropyridin-1(2*H*)-yl)ethyl)-3-(1*H*-indol-3-yl)pyrrolidine-2,5-dione (**6**)

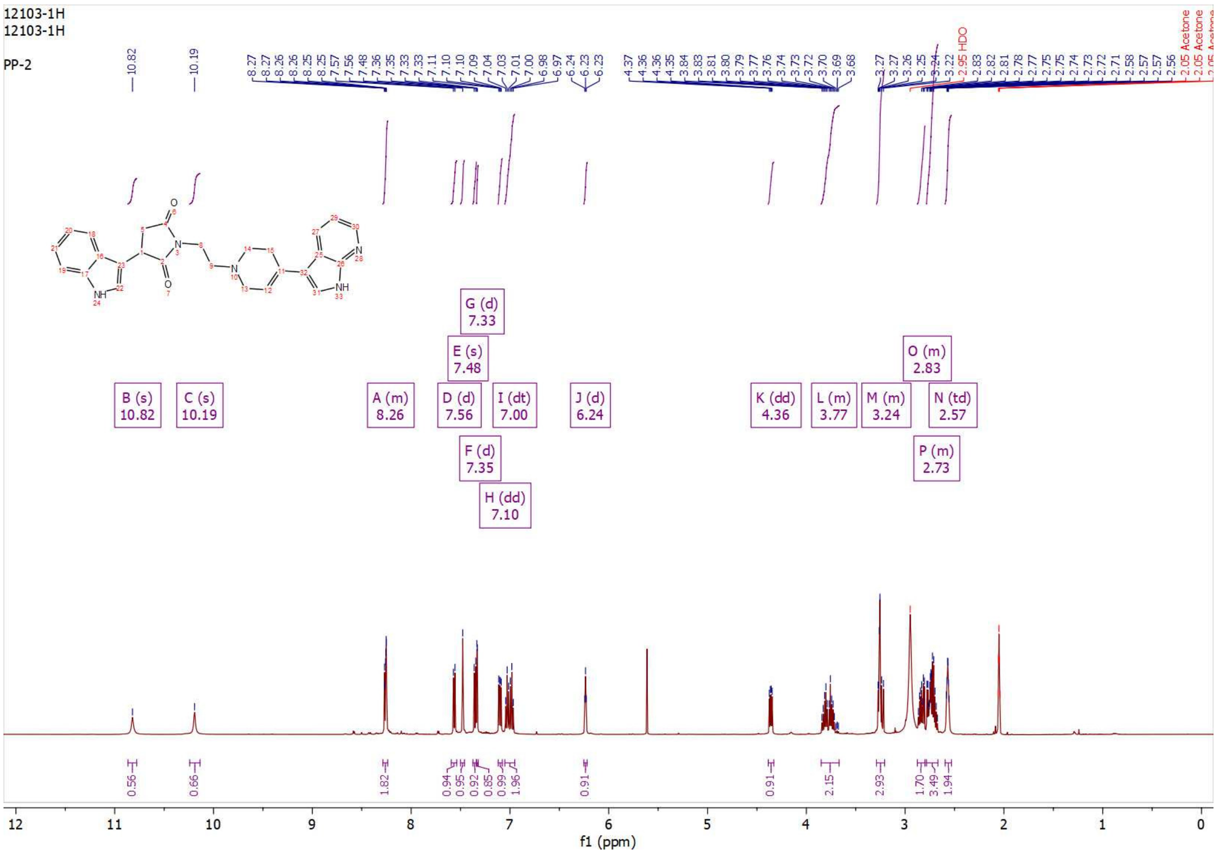

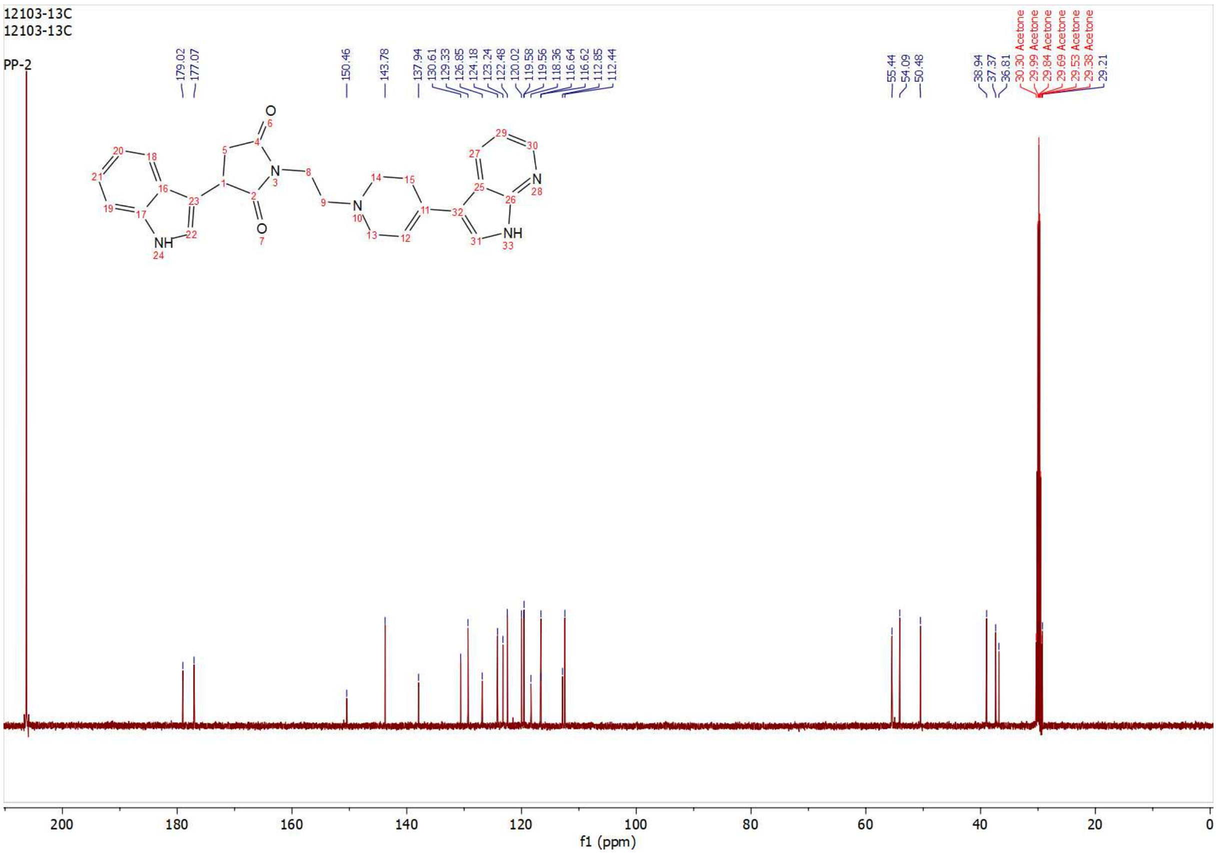

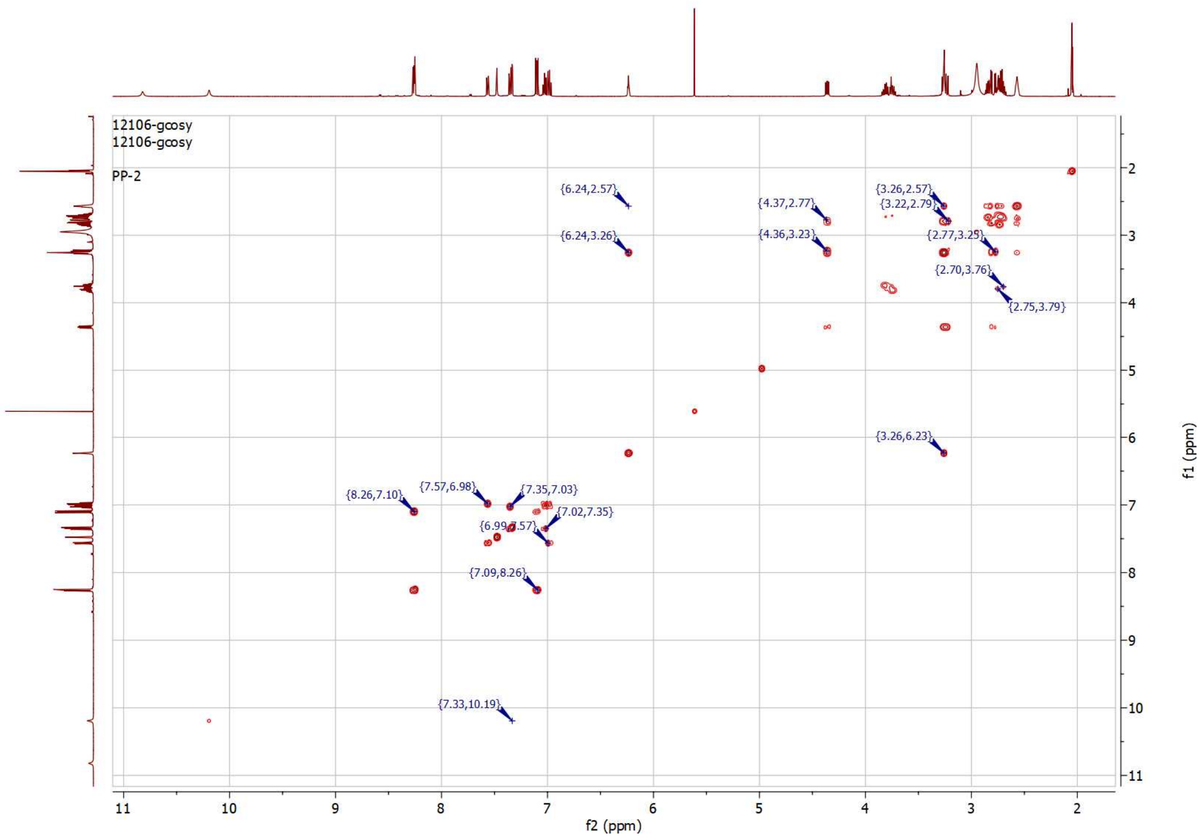
12106-gcosy
12106-gcosy
PP-2
{6.24,2.57}
{4.37,2.77}
{3.26,2.57}
{3.22,2.79}
{6.24,3.26}
{4.36,3.23}
{2.77,3.25}
{2.70,3.76}
{2.75,3.79}
{3.26,6.23}
{7.57,6.98}
{7.35,7.03}
{8.26,7.10}
{7.02,7.35}
{6.99,7.57}
{7.09,8.26}
{7.33,10.19}
f2 (ppm)
f1 (ppm)

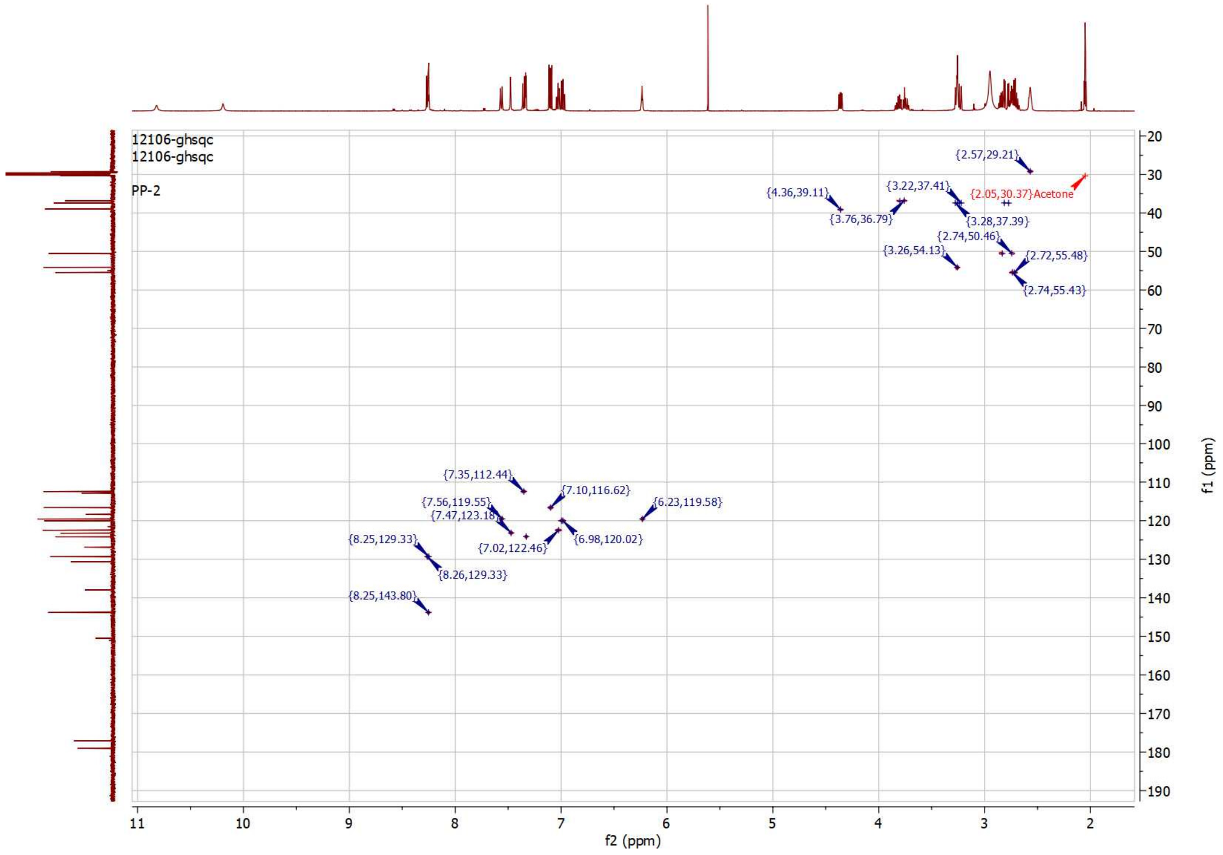
12106-ghsqc
12106-ghsqc
PP-2
{2.57,29.21}
{4.36,39.11}
{3.22,37.41}
{2.05,30.37} Acetone
{3.76,36.79}
{3.28,37.39}
{2.74,50.46}
{3.26,54.13}
{2.72,55.48}
{2.74,55.43}
{7.35,112.44}
{7.10,116.62}
{7.56,119.55}
{6.23,119.58}
{7.47,123.18}
{8.25,129.33}
{7.02,122.46}
{6.98,120.02}
{8.26,129.33}
{8.25,143.80}
f2 (ppm)
f1 (ppm)

6. HRMS spectra of 1-(2-(4-(1*H*-pyrrolo[2,3-*b*]pyridin-3-yl)-3,6-dihydropyridin-1(2*H*)-yl)ethyl)-3-(1*H*-indol-3-yl)pyrrolidine-2,5-dione (**6**)

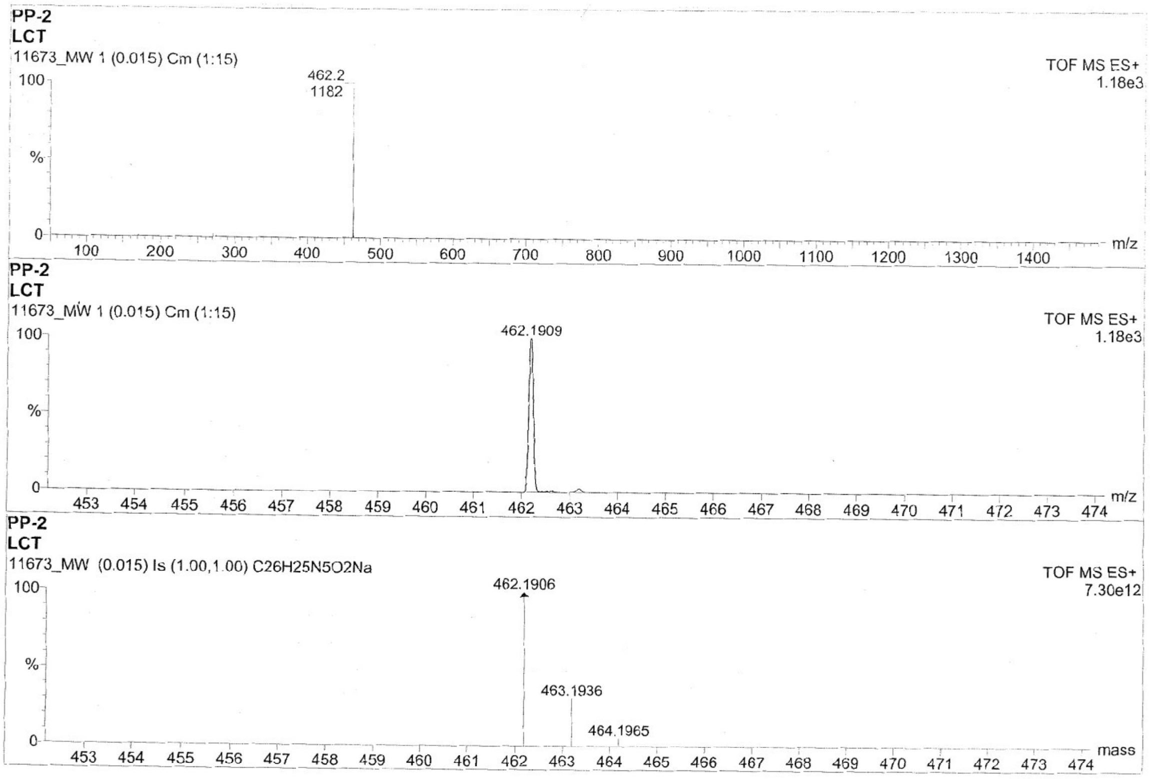

7. ^{1}H and ^{13}C NMR spectra of 1-(4-(4-(1*H*-pyrrolo[2,3-*b*]pyridin-3-yl)-3,6-dihydropyridin-1(2*H*)-yl)butyl)-3-(5-methoxy-1*H*-indol-3-yl)pyrrolidine-2,5-dione (**7**)

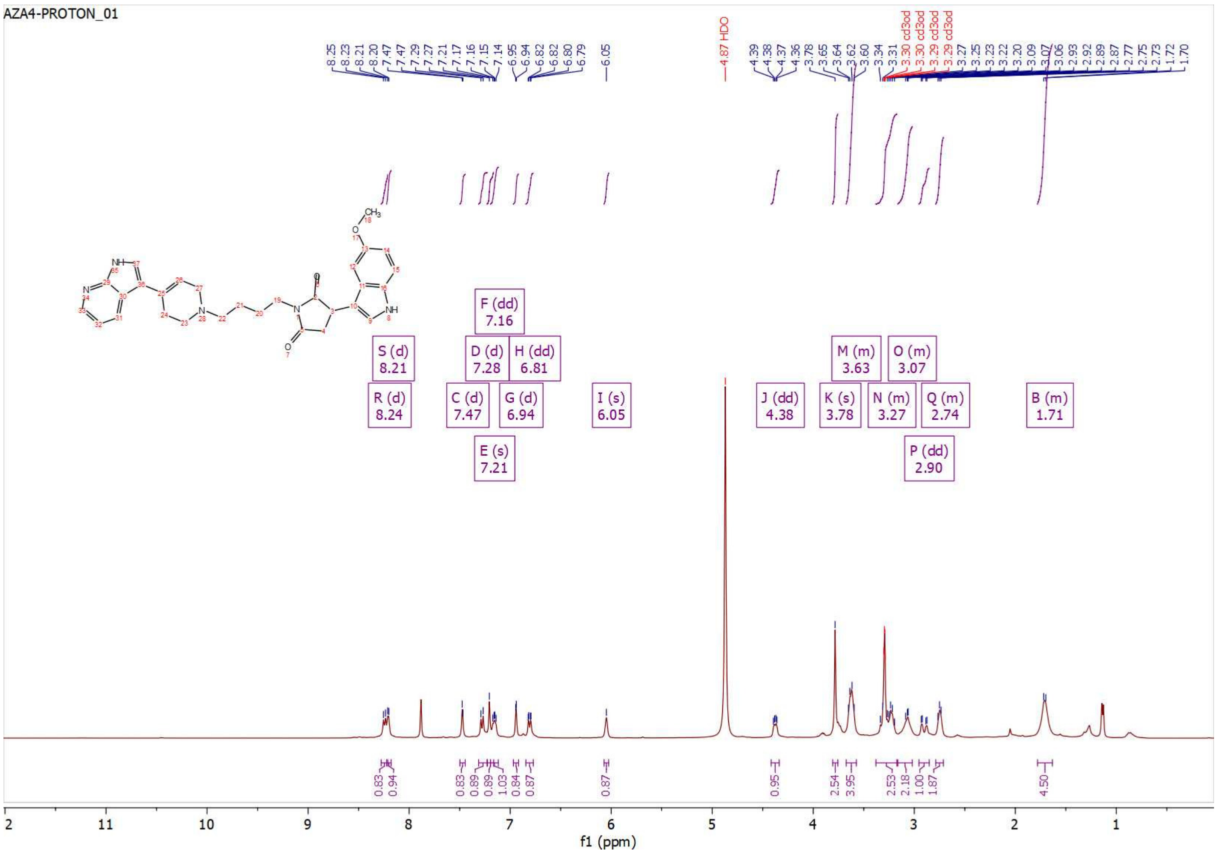

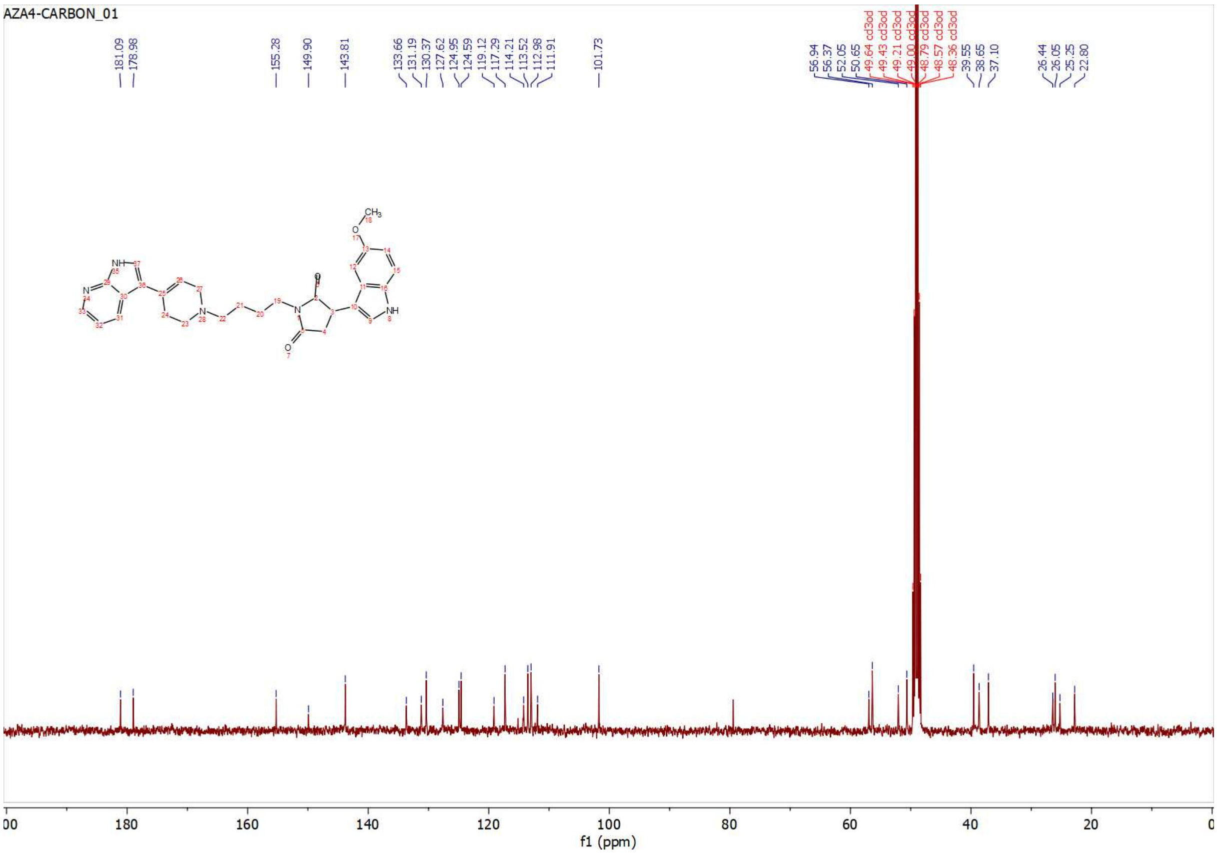

8. HRMS spectra of 1-(4-(4-(1*H*-pyrrolo[2,3-*b*]pyridin-3-yl)-3,6-dihydropyridin-1(2*H*)-yl)butyl)-3-(5-methoxy-1*H*-indol-3-yl)pyrrolidine-2,5-dione (**7**)

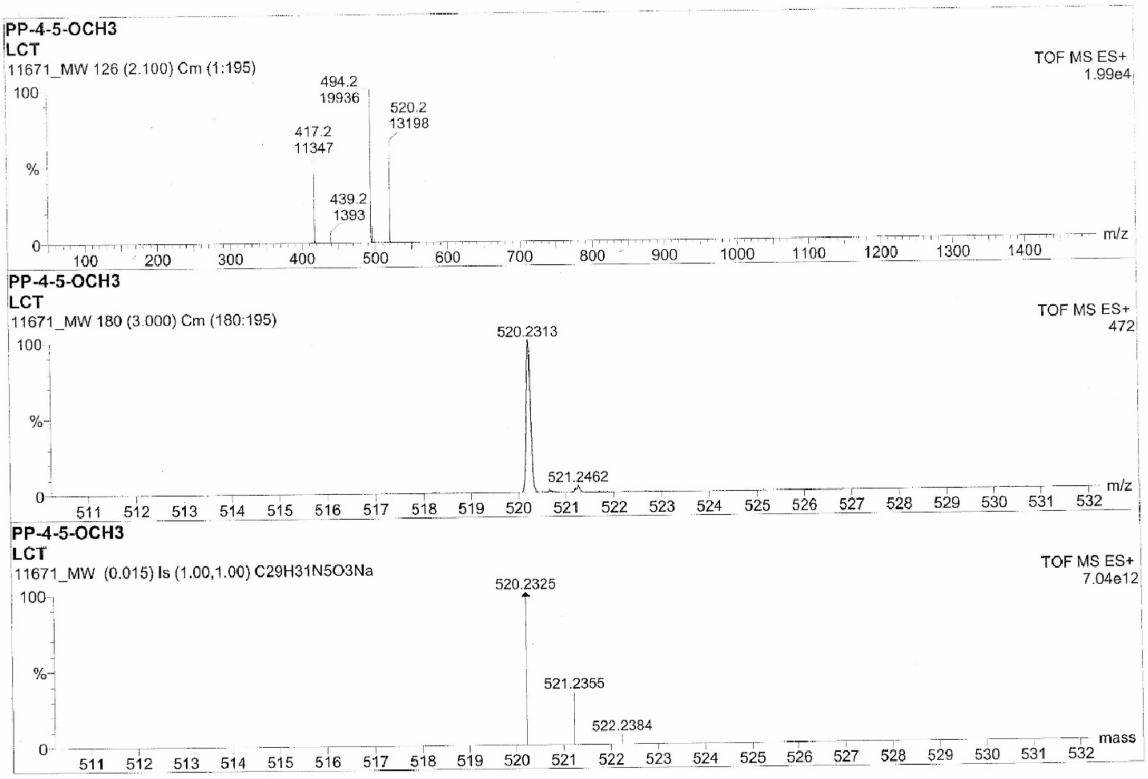

9. ^{1}H and ^{13}C NMR spectra of 1-(3-(4-(1*H*-pyrrolo[2,3-*b*]pyridin-3-yl)-3,6-dihydropyridin-1(2*H*)-yl)propyl)-3-(5-methoxy-1*H*-indol-3-yl)pyrrolidine-2,5-dione (**8**)

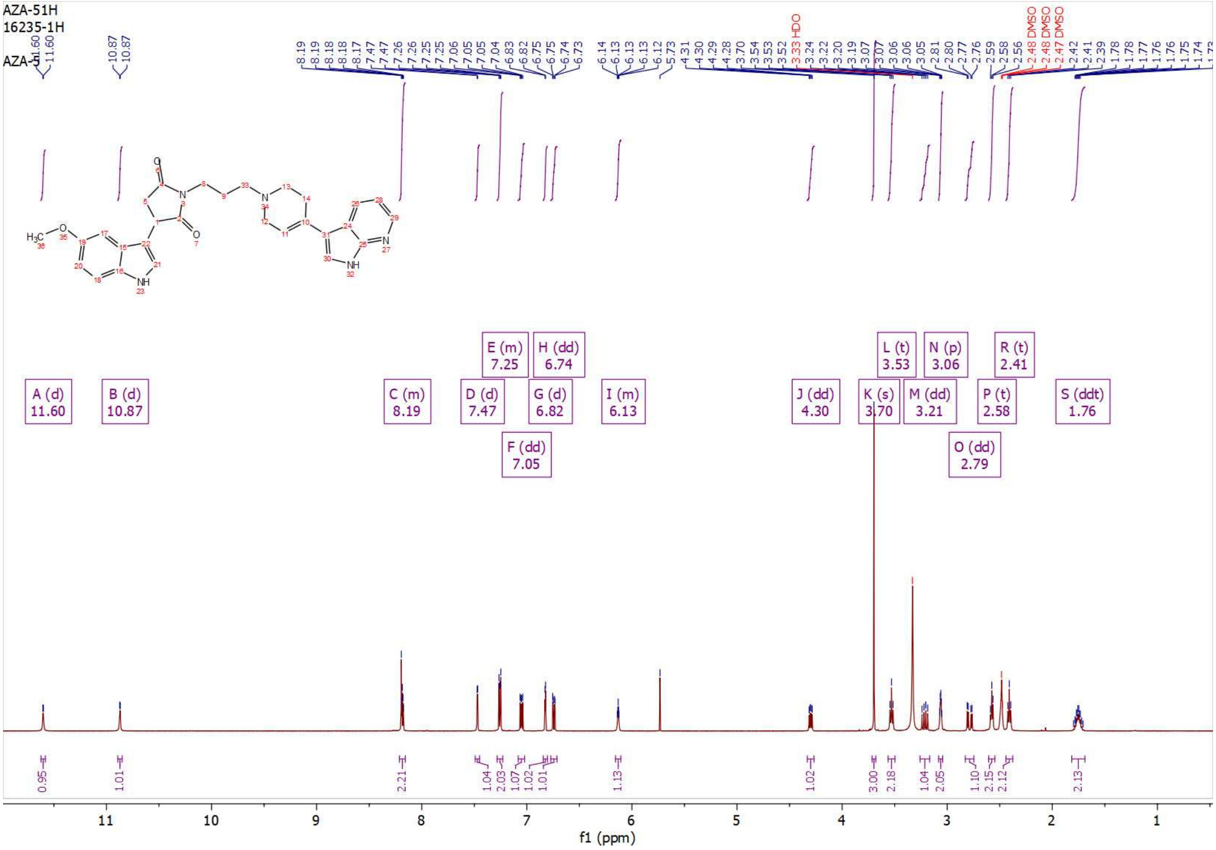

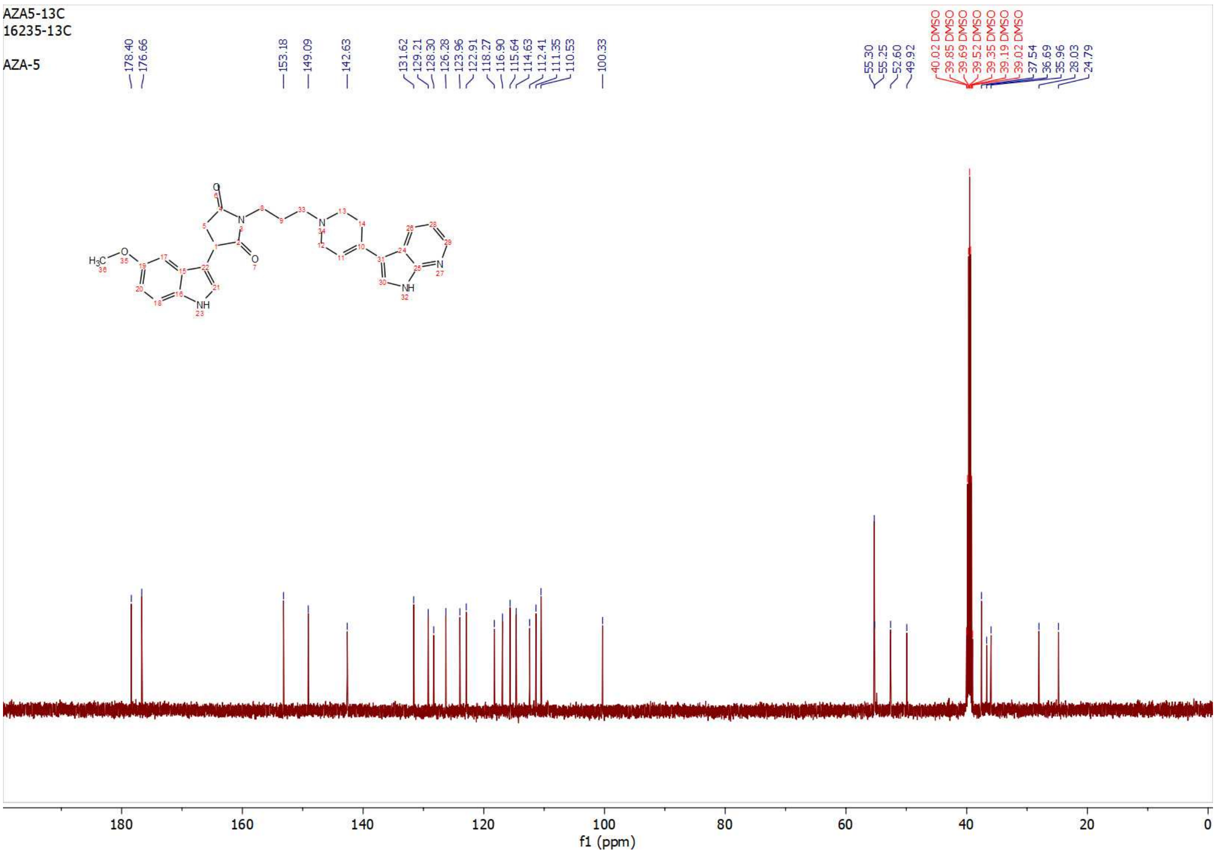

10. HRMS spectra of 1-(3-(4-(1*H*-pyrrolo[2,3-*b*]pyridin-3-yl)-3,6-dihydropyridin-1(2*H*)-yl)propyl)-3-(5-methoxy-1*H*-indol-3-yl)pyrrolidine-2,5-dione (**8**)

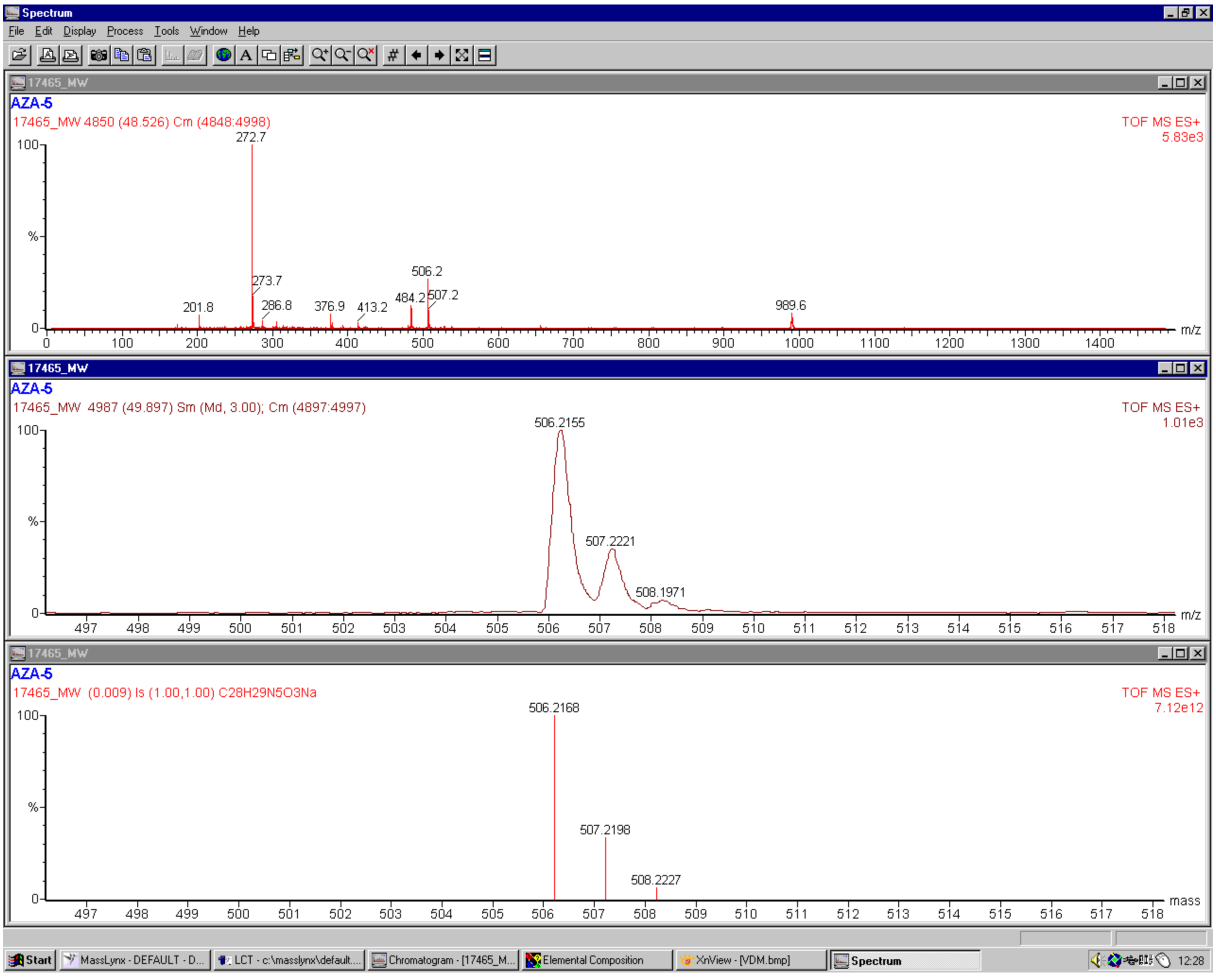

11. ^{1}H and ^{13}C NMR spectra of 1-(2-(4-(1*H*-pyrrolo[2,3-*b*]pyridin-3-yl)-3,6-dihydropyridin-1(2*H*)-yl)ethyl)-3-(5-methoxy-1H-indol-3-yl)pyrrolidine-2,5-dione (**9**)

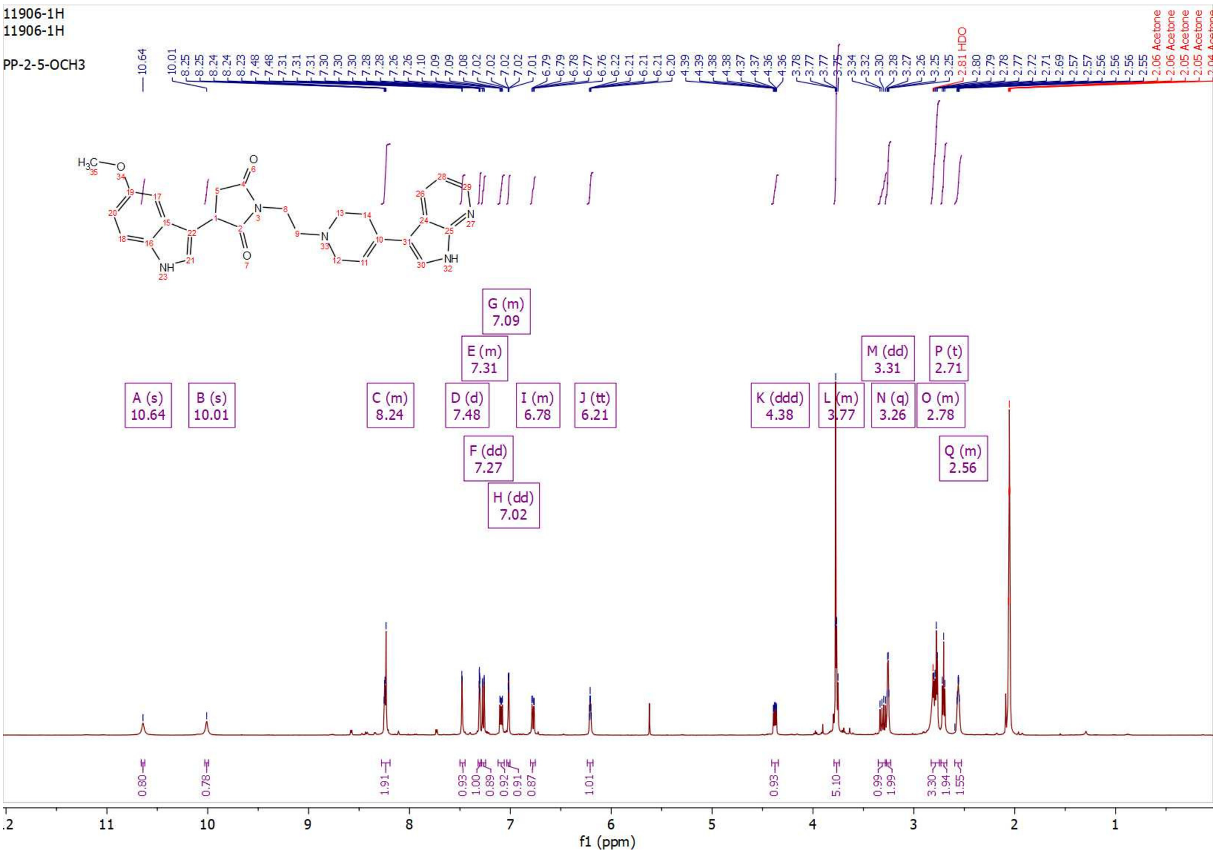

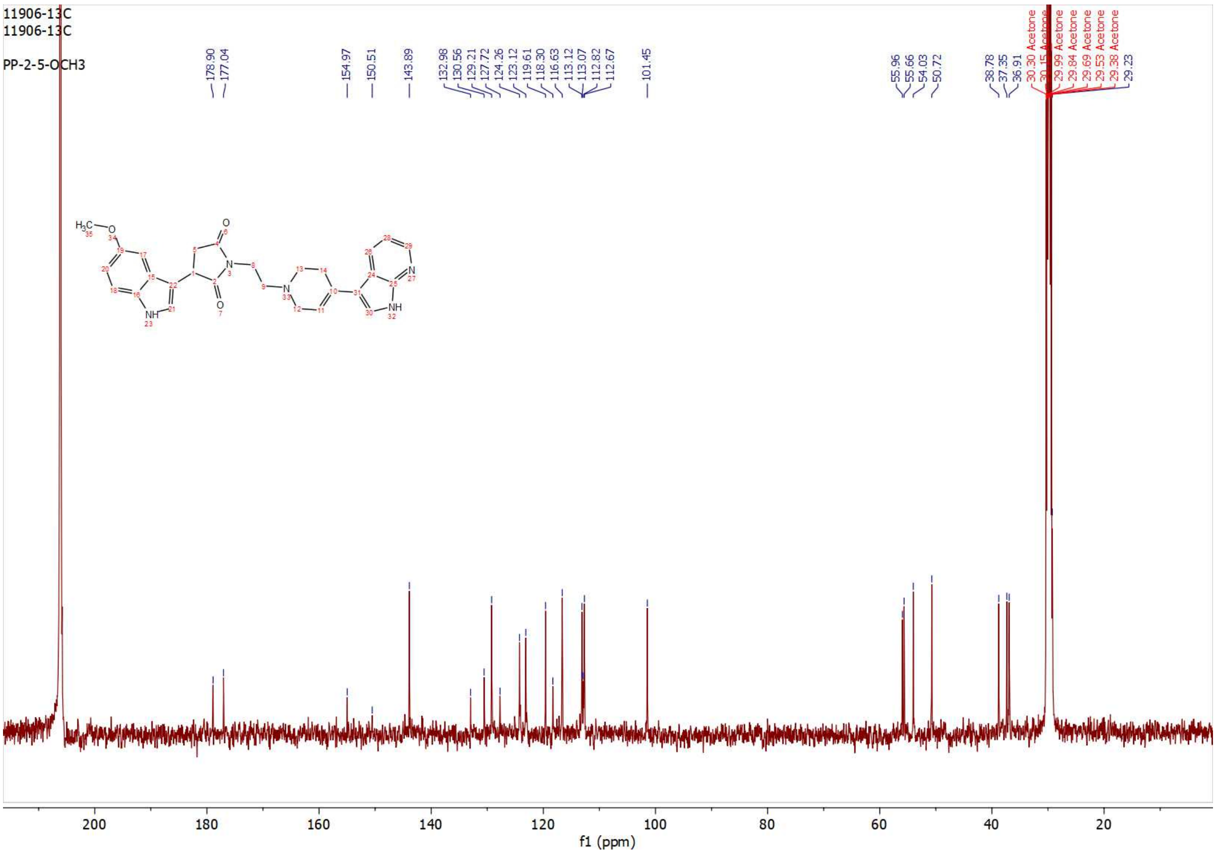

12. HRMS spectra of 1-(2-(4-(1*H*-pyrrolo[2,3-*b*]pyridin-3-yl)-3,6-dihydropyridin-1(2*H*)-yl)ethyl)-3-(5-methoxy-1H-indol-3-yl)pyrrolidine-2,5-dione (**9**)

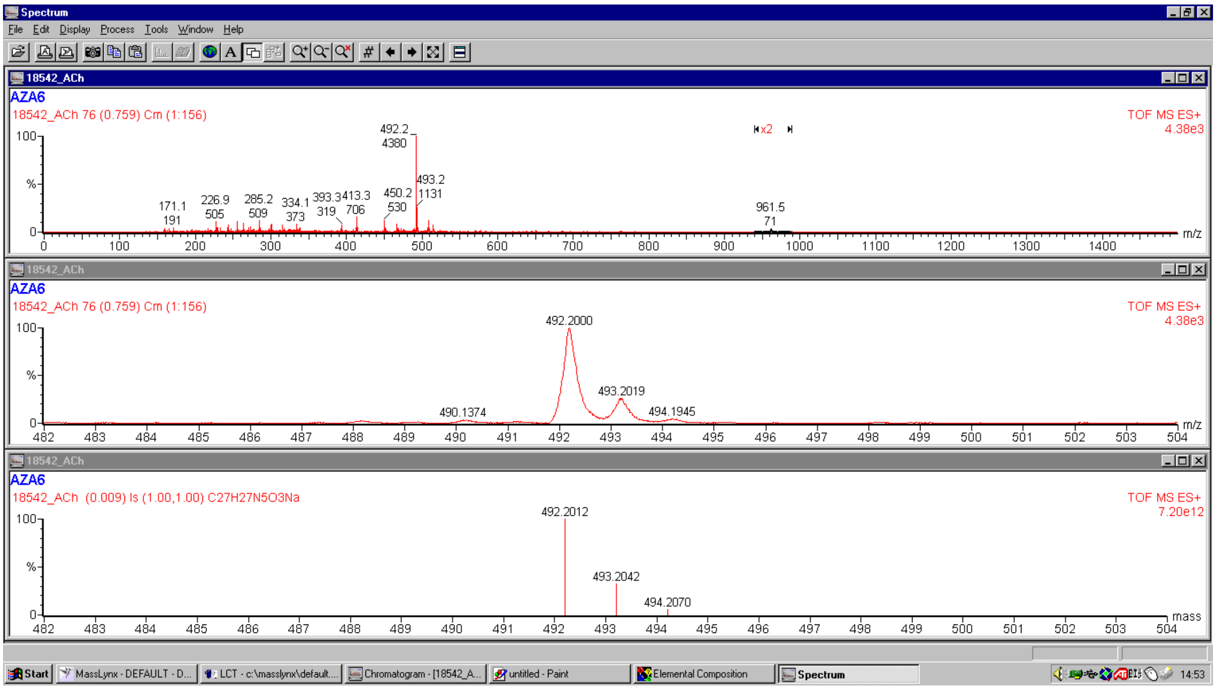

13. ^{1}H and ^{13}C NMR spectra of 1-(4-(4-(1*H*-pyrrolo[2,3-*b*]pyridin-3-yl)-3,6-dihydropyridin-1(2*H*)-yl)butyl)-3-(5-fluoro-1*H*-indol-3-yl)pyrrolidine-2,5-dione (**10**)

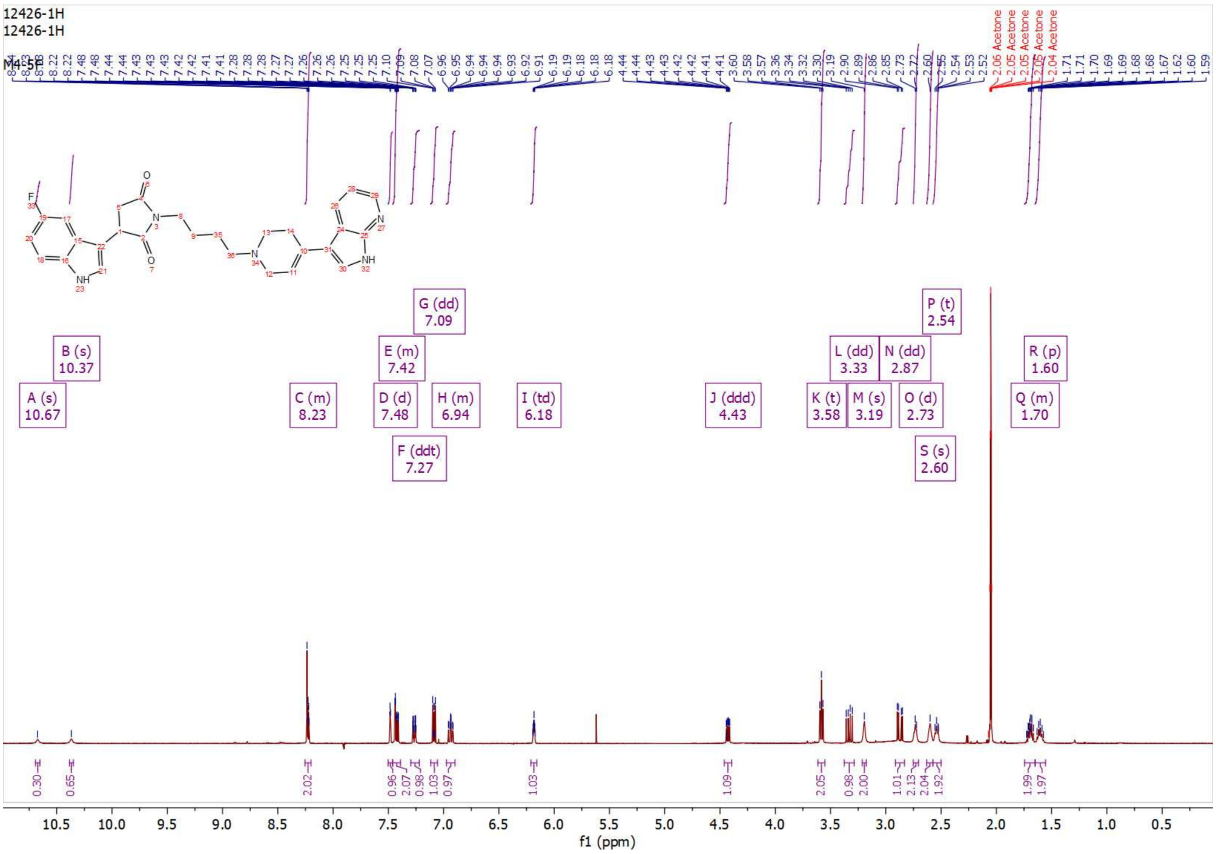

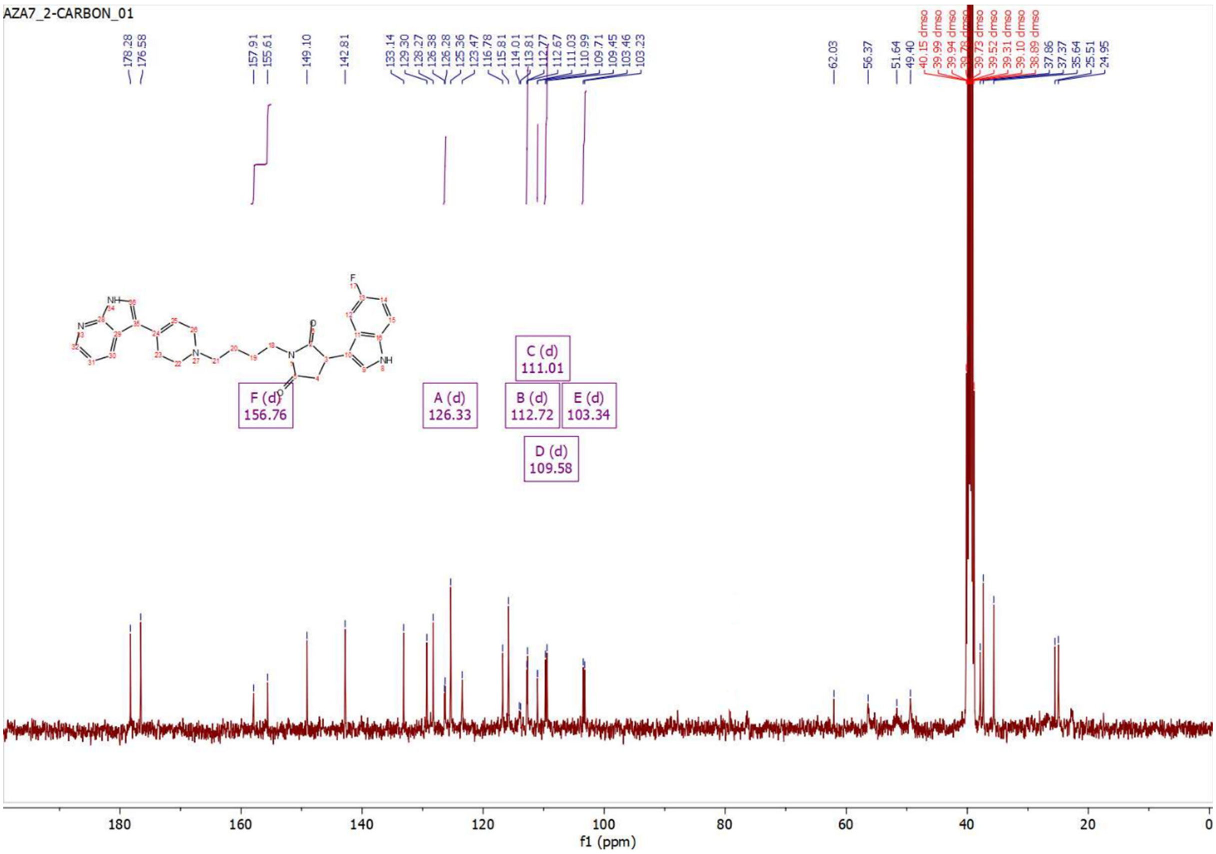

14. HRMS spectra of 1-(4-(4-(1*H*-pyrrolo[2,3-*b*]pyridin-3-yl)-3,6-dihydropyridin-1(2*H*)-yl)butyl)-3-(5-fluoro-1*H*-indol-3-yl)pyrrolidine-2,5-dione (**10**)

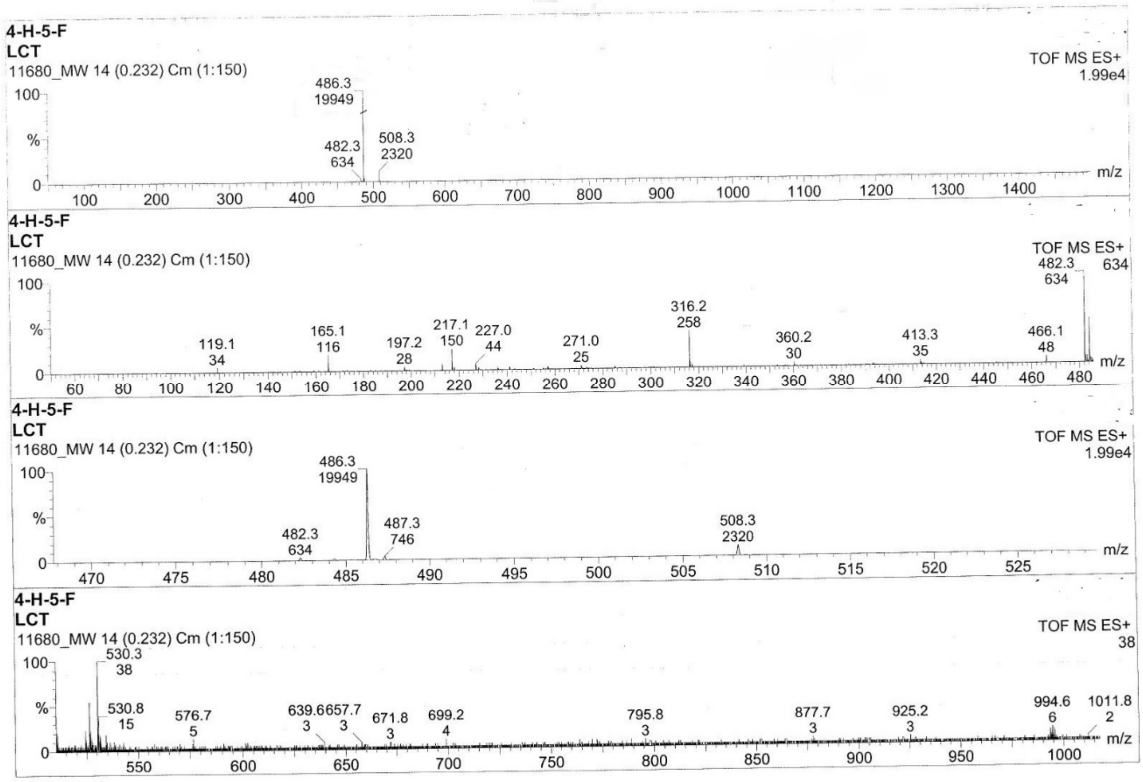

15. ^{1}H and ^{13}C NMR spectra of 1-(3-(4-(1*H*-pyrrolo[2,3-*b*]pyridin-3-yl)-3,6-dihydropyridin-1(2*H*)-yl)propyl)-3-(5-fluoro-1*H*-indol-3-yl)pyrrolidine-2,5-dione (**11**)

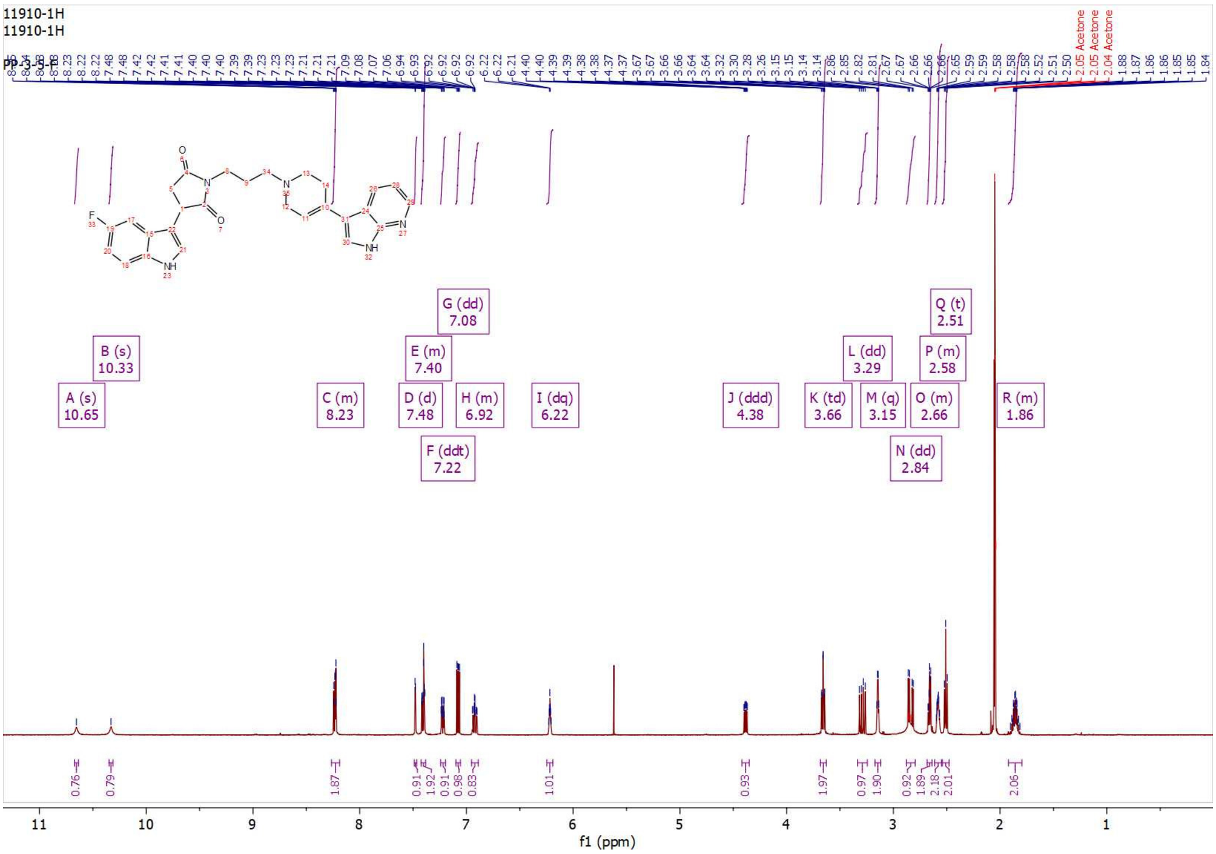

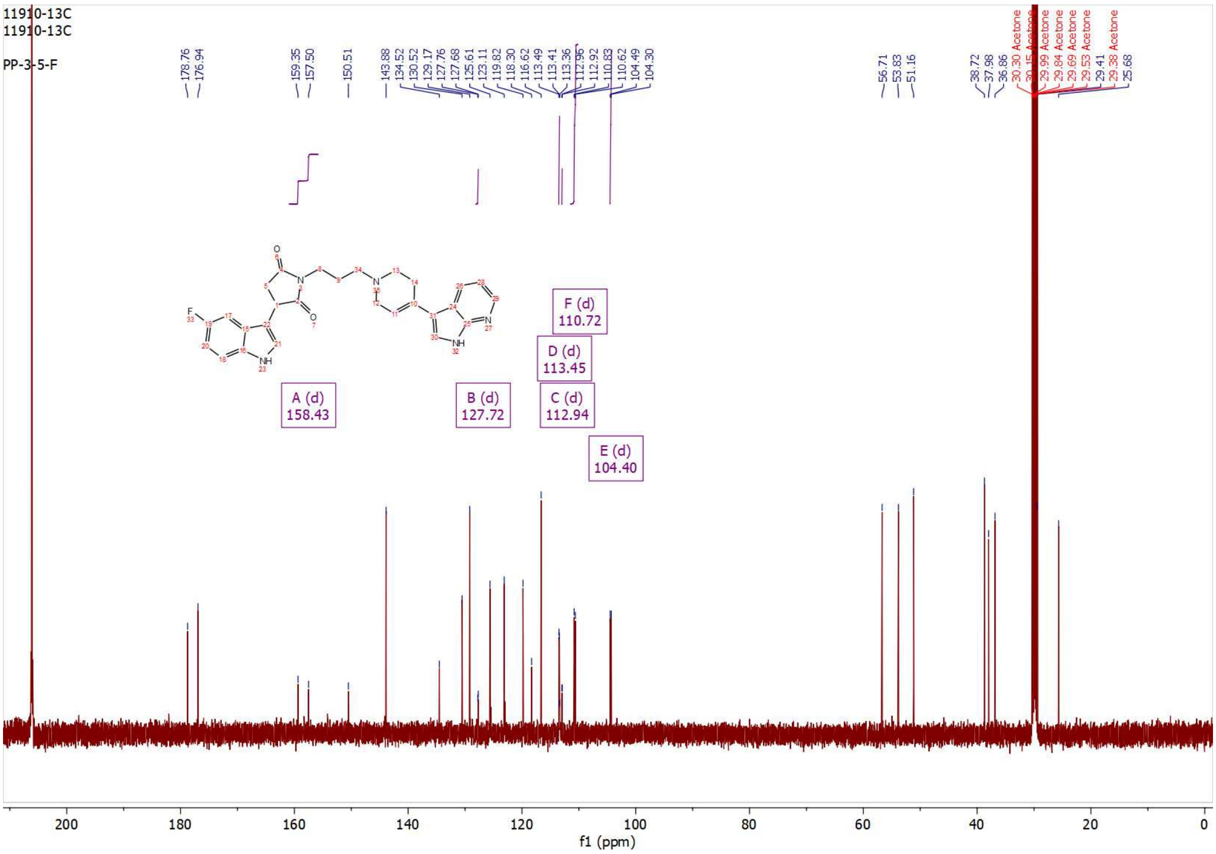

16. HRMS spectra of 1-(3-(4-(1*H*-pyrrolo[2,3-*b*]pyridin-3-yl)-3,6-dihydropyridin-1(2*H*)-yl)propyl)-3-(5-fluoro-1*H*-indol-3-yl)pyrrolidine-2,5-dione (**11**)

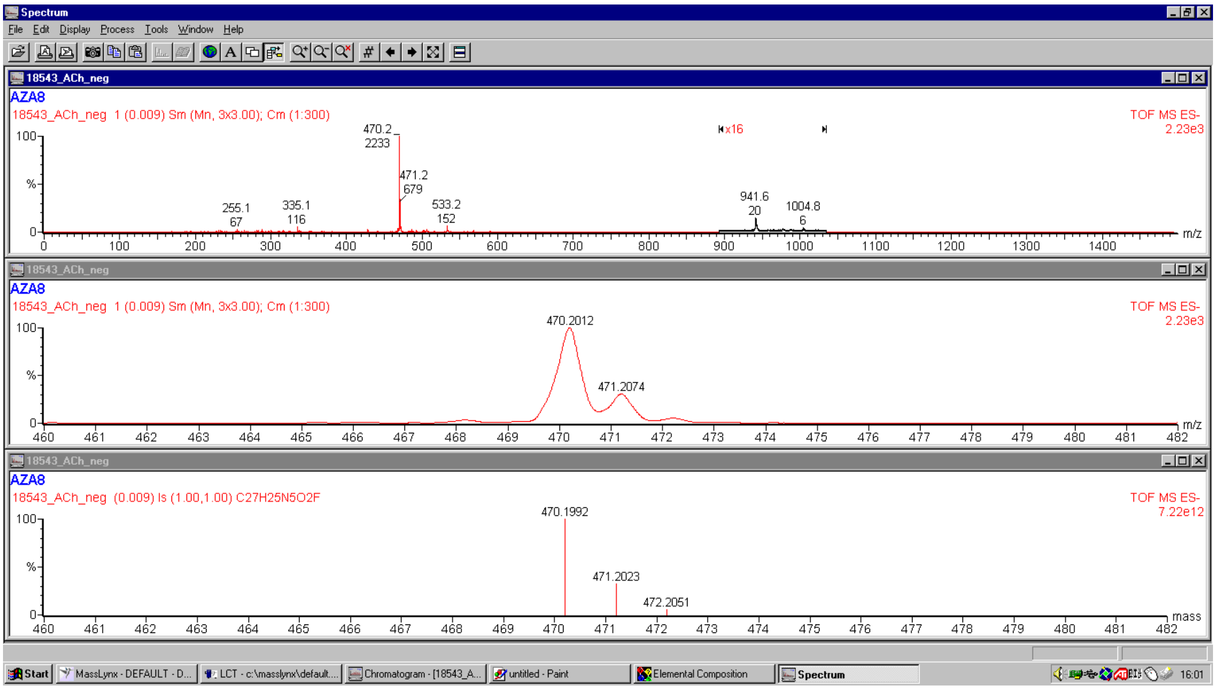

17. ^1H and ^{13}C NMR spectra of 1-(2-(4-(1*H*-pyrrolo[2,3-*b*]pyridin-3-yl)-3,6-dihydropyridin-1(2*H*)-yl)ethyl)-3-(5-fluoro-1*H*-indol-3-yl)pyrrolidine-2,5-dione (**12**)

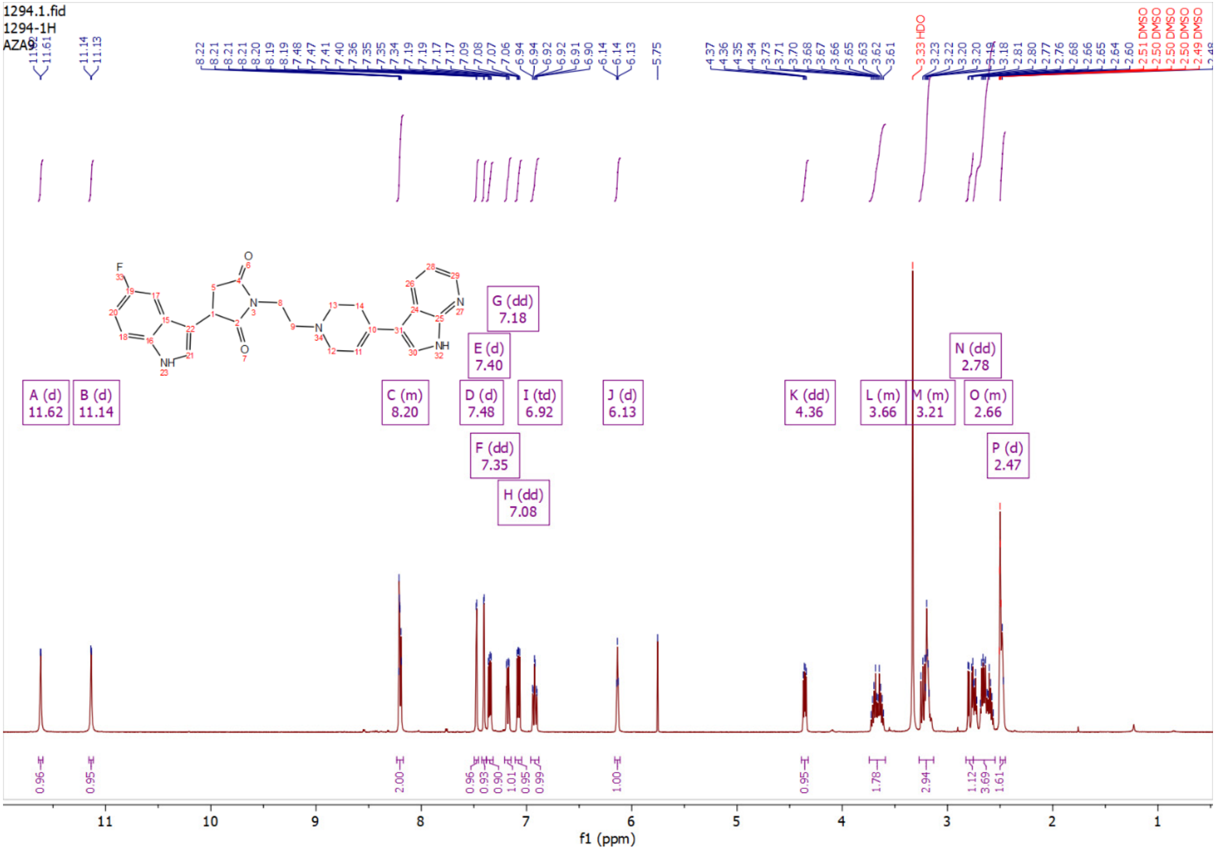

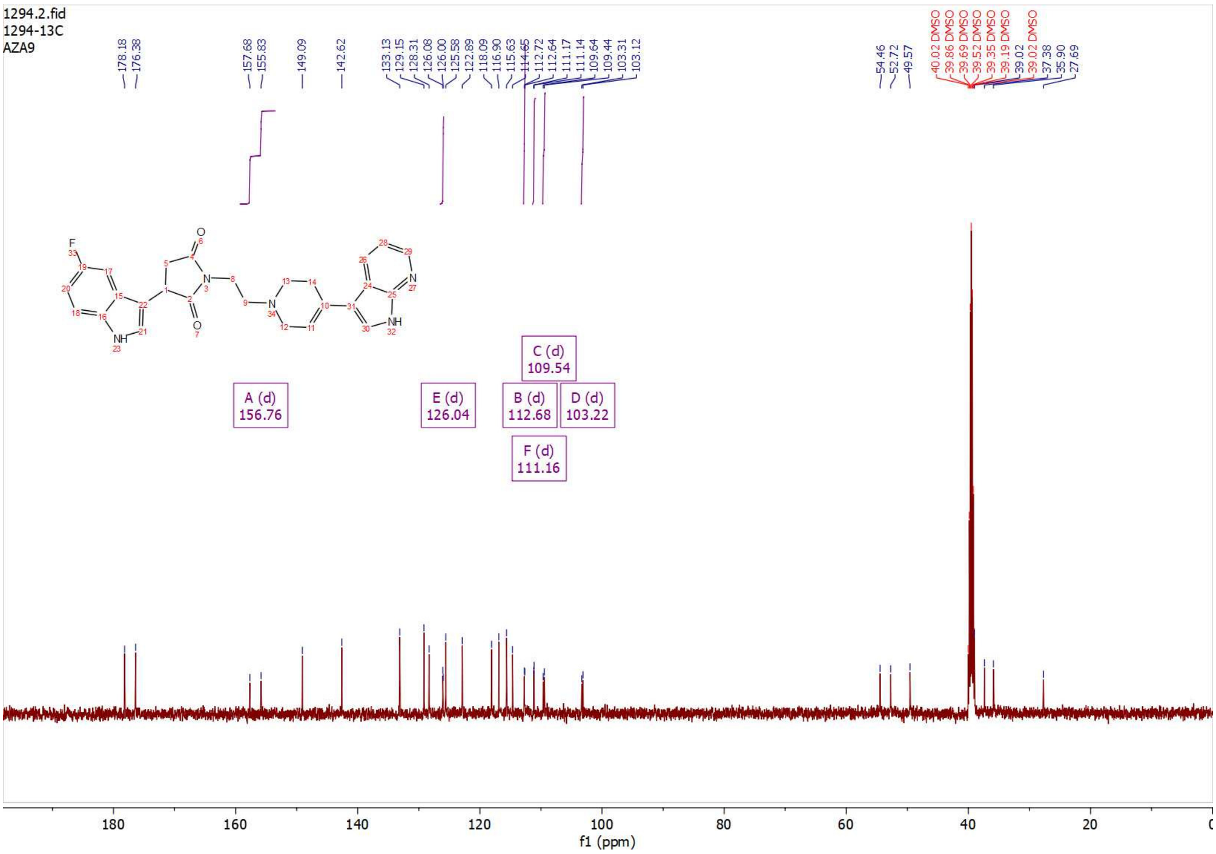

18. HRMS spectra of 1-(2-(4-(1*H*-pyrrolo[2,3-*b*]pyridin-3-yl)-3,6-dihydropyridin-1(2*H*)-yl)ethyl)-3-(5-fluoro-1*H*-indol-3-yl)pyrrolidine-2,5-dione (**12**)

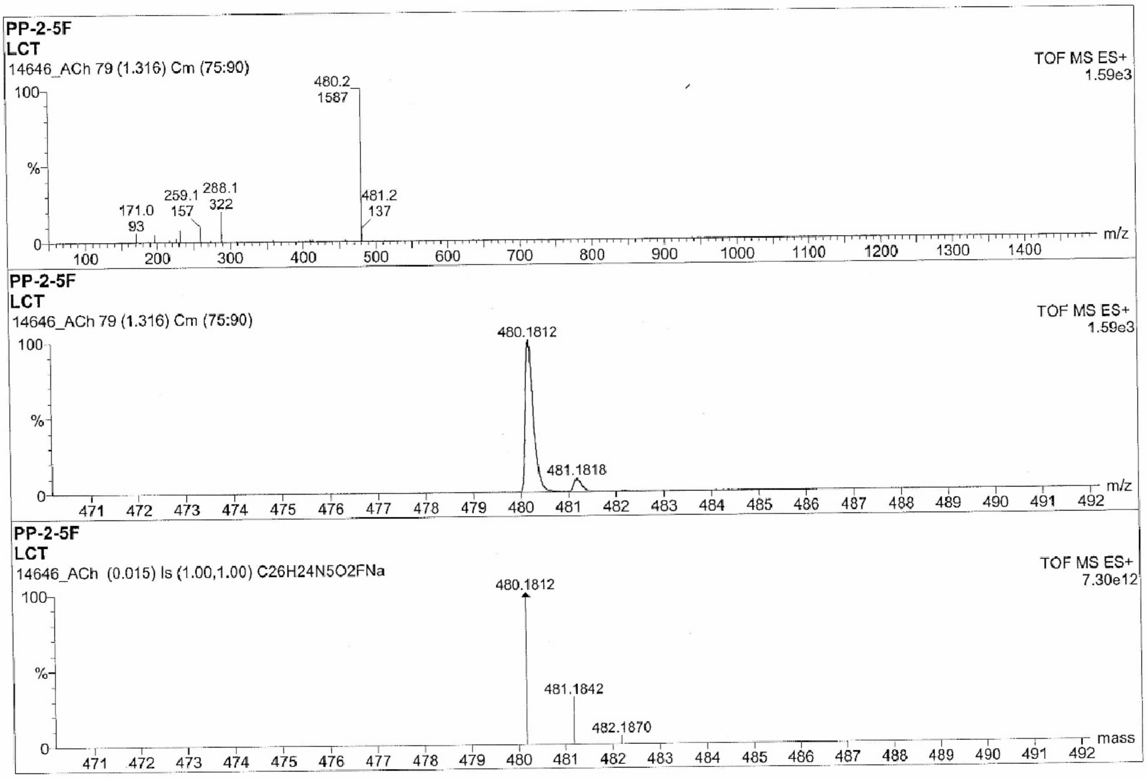

Supplement: Supplementary file 1 [file ijms-25-11276-s001.zip › ijms-3250778-SI.pdf]
